# Supplementary material for: Noncanonical roles of chemokine regions in CCR9 activation revealed by structural modeling and mutational mapping
Source: Nat Commun. 2025 Aug 18;16:7695. doi: 10.1038/s41467-025-62321-9 (PMC12361432; doi:10.1038/s41467-025-62321-9)
Supplement: Supplementary file 1 — Supplementary Information [file 41467_2025_62321_MOESM1_ESM.pdf]

Supplementary Figures and Legends

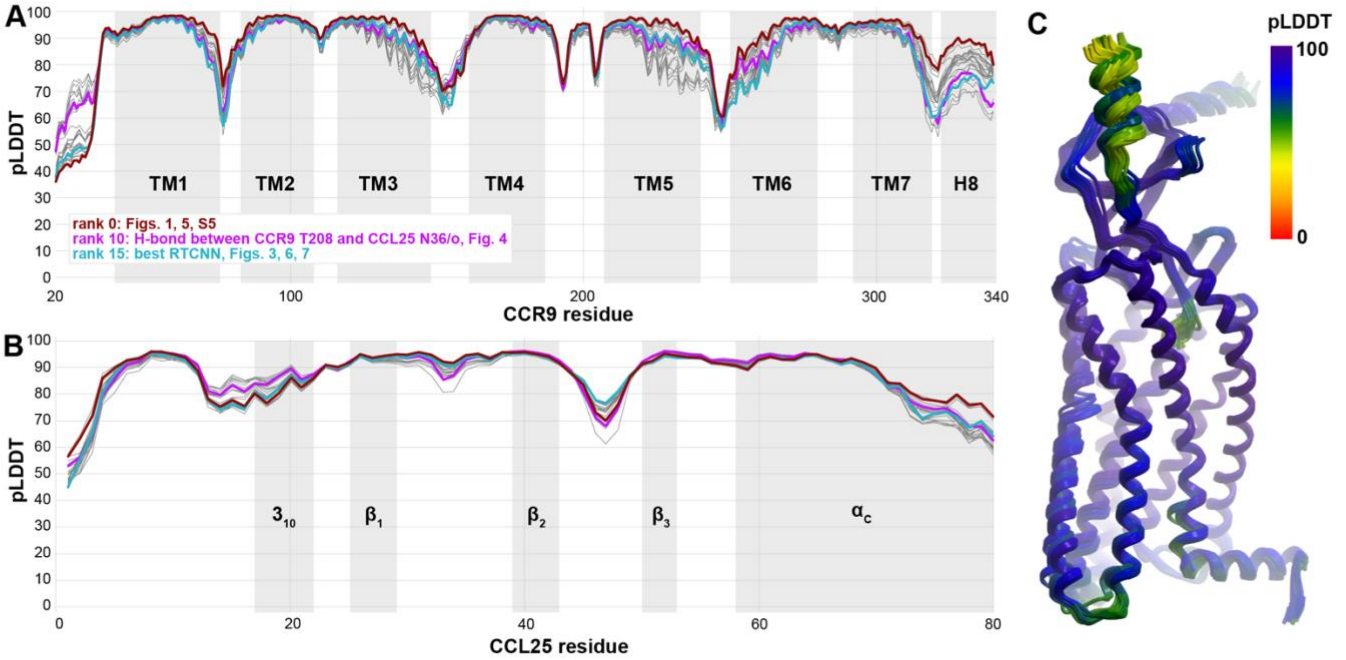

Supplementary Figure 1. Prediction confidence for the 5x5 AF2 models of the CCR9-CCL25 complex.

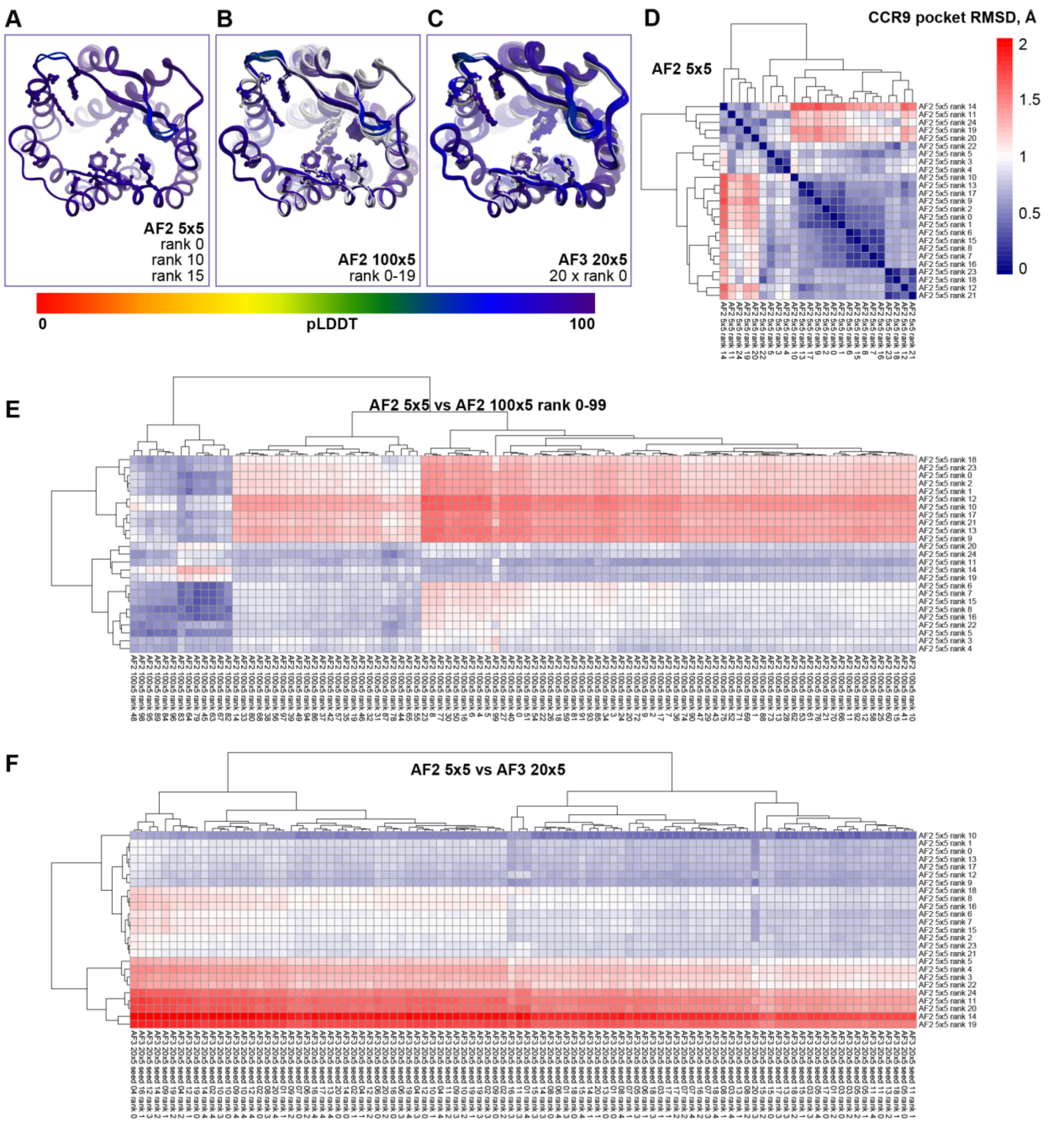

Supplementary Figure 2. Conformational variation and prediction confidence (pLDDT scores) for the key CCR9 CRS2 residues in the various models of the CCR9-CCL25 complex constructed in this work.
(**A-C**) Conformational variation for the 3 models (selected for manuscript analyses) from the original 5x5 AF2 ensemble (**A**), 20 most confident models from the extended 100x5 AF2 ensemble (**B**), and 20 rank 0 models from the 20x5 AF3 ensemble (**C**). Pockets are viewed perpendicular to the plane of the membrane from the extracellular side. Sticks denote residues with largest chemokine contacts as shown in **Supplementary Fig. 6**. Ribbons and sticks are colored by pLDDT. In (**B**) and (**C**), the three models from (**A**) are shown in white for reference. (**D**) Pairwise pocket RMSD between the models in the original 5x5 AF2 ensemble. (**E**) Pairwise pocket RMSD between the models in the original 5x5 AF2 ensemble and the first 100 models from the extended 100x5 AF2 ensemble. (**F**)

Pairwise pocket RMSD between the models in the original 5x5 AF2 ensemble and the 20x5 AF3 ensemble. In (**D-F**), RMSD is measured on side-chain non-hydrogen atoms of CCR9 residues with strongest contacts as shown in **Supplementary Fig. 6**.

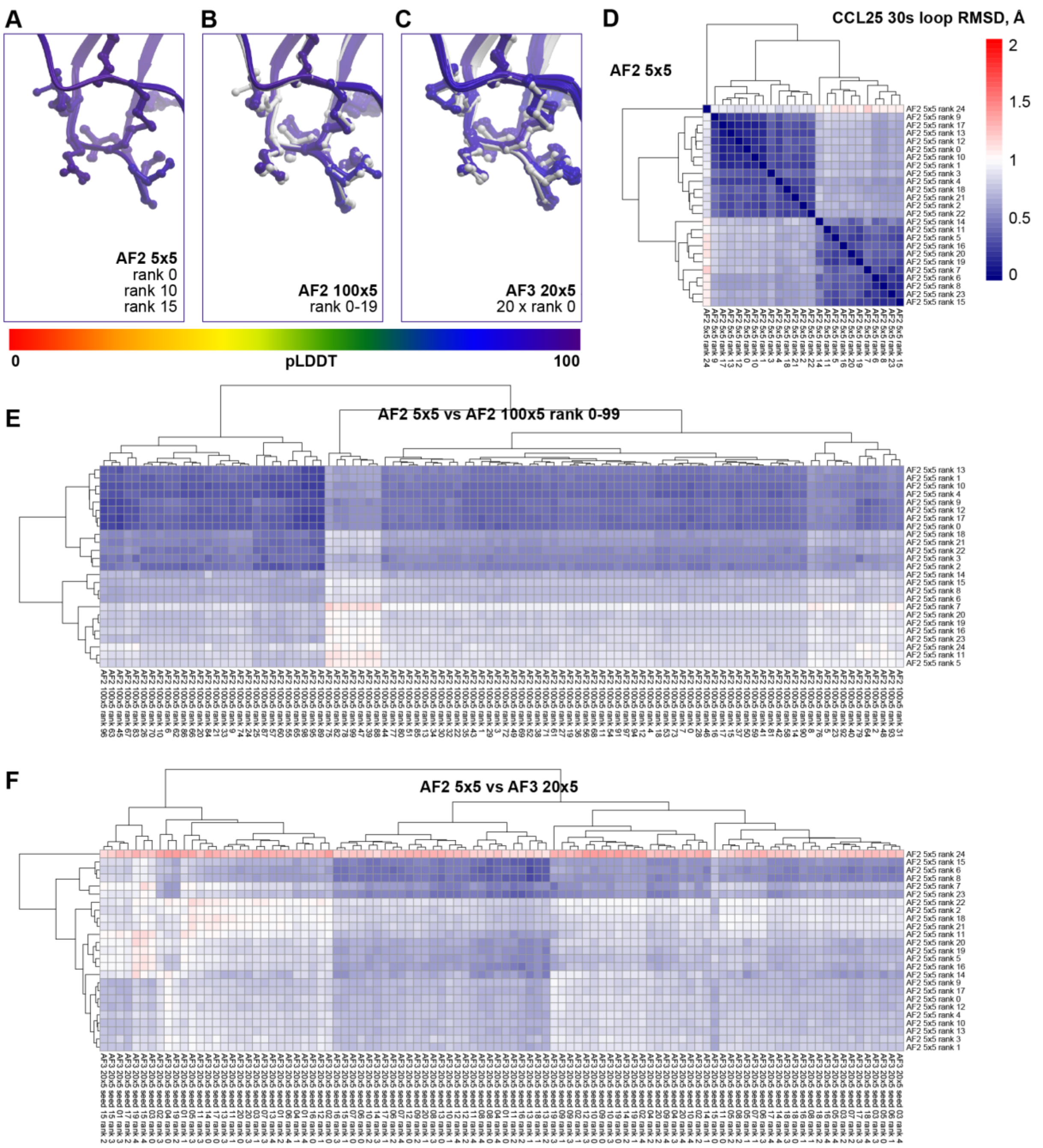

Supplementary Figure 3. Conformational variation and prediction confidence (pLDDT scores) for the CCL25 30s loop in various models of the CCR9-CCL25 complex constructed in this work.

(**A-C**) Conformational variation for the 3 models (selected for manuscript analyses) from the original 5x5 AF2 ensemble (**A**), 20 most confident models from the extended 100x5 AF2 ensemble (**B**), and 20 rank 0 models from the 20x5 AF3 ensemble (**C**). Chemokine loops are viewed parallel to the plane of the membrane; sticks denote CCL25 residues 30-37. Ribbons and sticks are colored by pLDDT. In (**B**) and (**C**), the three models from (**A**) are shown in white for reference. (**D**) Pairwise pocket-aligned chemokine 30s loop RMSD between the models in the original 5x5 AF2 ensemble. (**E**) Pairwise pocket-aligned chemokine 30s loop RMSD between the models in the original 5x5

AF2 ensemble and the first 100 models from the extended 100x5 AF2 ensemble. (**F**) Pairwise pocket-aligned chemokine 30s loop RMSD between the models in the original 5x5 AF2 ensemble and the 20x5 AF3 ensemble.
In (**D-F**), RMSD is measured on all non-hydrogen atoms of CCL25 residues 30-37 following CCR9 pocket superposition as in **Supplementary Fig. 2**.

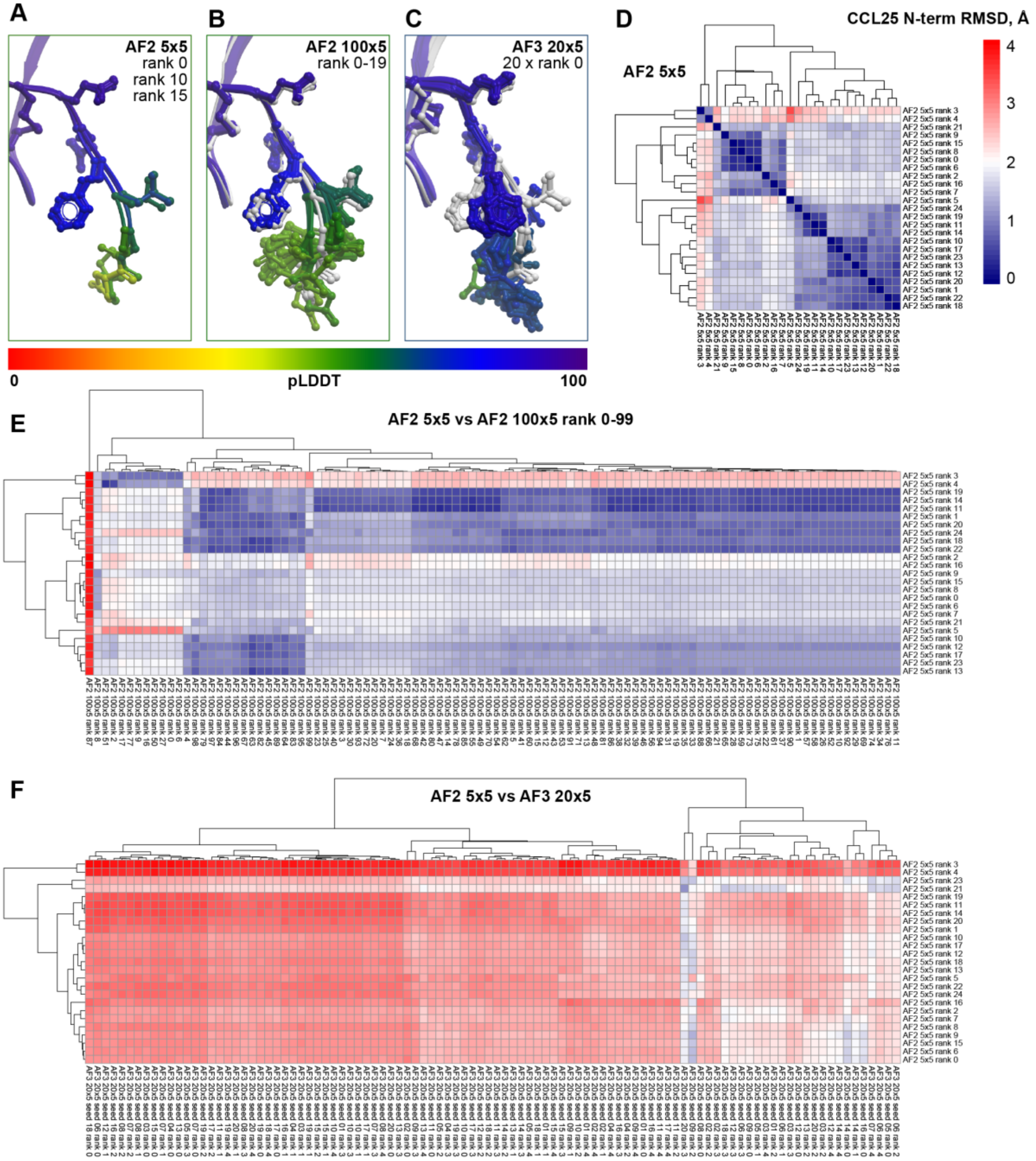

Supplementary Figure 4. Conformational variation and prediction confidence (pLDDT scores) for the CCL25 N-terminus in various models of the CCR9-CCL25 complex constructed in this work.

(**A-C**) Conformational variation for the 3 models (selected for manuscript analyses) from the original 5x5 AF2 ensemble (**A**), 20 most confident models from the extended 100x5 AF2 ensemble (**B**), and 20 rank 0 models from the 20x5 AF3 ensemble (**C**). Chemokine N-termini are viewed parallel to the plane of the membrane; sticks denote CCL25 residues 1-6. Ribbons and sticks are colored by pLDDT. In (**B**) and (**C**), the three models from (**A**) are shown in white for reference. (**D**) Pairwise pocket-aligned chemokine N-terminus RMSD between the models in the original 5x5 AF2 ensemble. (**E**) Pairwise pocket-aligned chemokine N-terminus RMSD between the models in the original 5x5 AF2 ensemble and the first 100 models from the extended 100x5 AF2 ensemble. (**F**) Pairwise pocket-

aligned chemokine N-terminus RMSD between the models in the original 5x5 AF2 ensemble and the 20x5 AF3 ensemble. In (**D-F**), RMSD is measured on all non-hydrogen atoms of CCL25 residues 1-6 following CCR9 pocket superposition as in **Supplementary Fig. 2**.

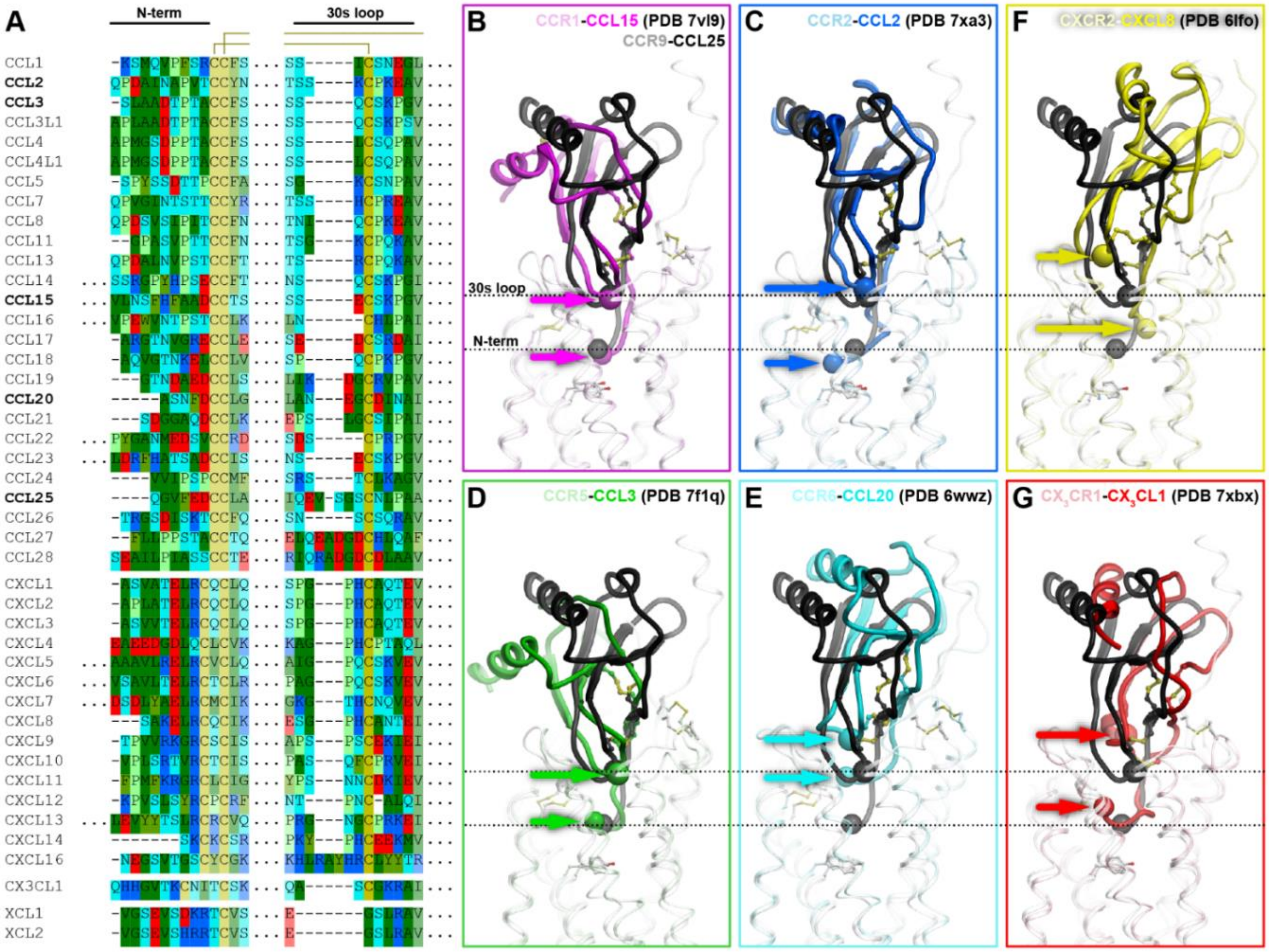

Supplementary Figure 5. Comparison of sequences and binding modes of CCL25 with other chemokines.

(**A**) Partial sequence alignment of N-termini and 30s loops of human chemokines from the CC, CXC, CX3C, and XC subfamilies. Amino acid letters are colored by function (positive, negative, or neutral polar residues are highlighted in blue, red, and cyan respectively; non-polar residues in green, proline in light green, and cysteine in yellow). Chemokines whose experimental structures are shown in (**B-G**) are bold. (**B-G**) Superimpositions of the predicted CCR9-CCL25 complex (grey and black ribbons) with selected experimental structures of other chemokine-receptor complexes (colored ribbons). Complexes are superimposed by receptor TM domains to depict variation in binding depths of chemokine N-termini and 30s loops (colored arrows) in reference to those of CCL25 (dashed lines). For each chemokine structure shown, Cα positions of the N-terminal residue and the residue preceding the third (30s loop) cysteine are denoted with spheres.

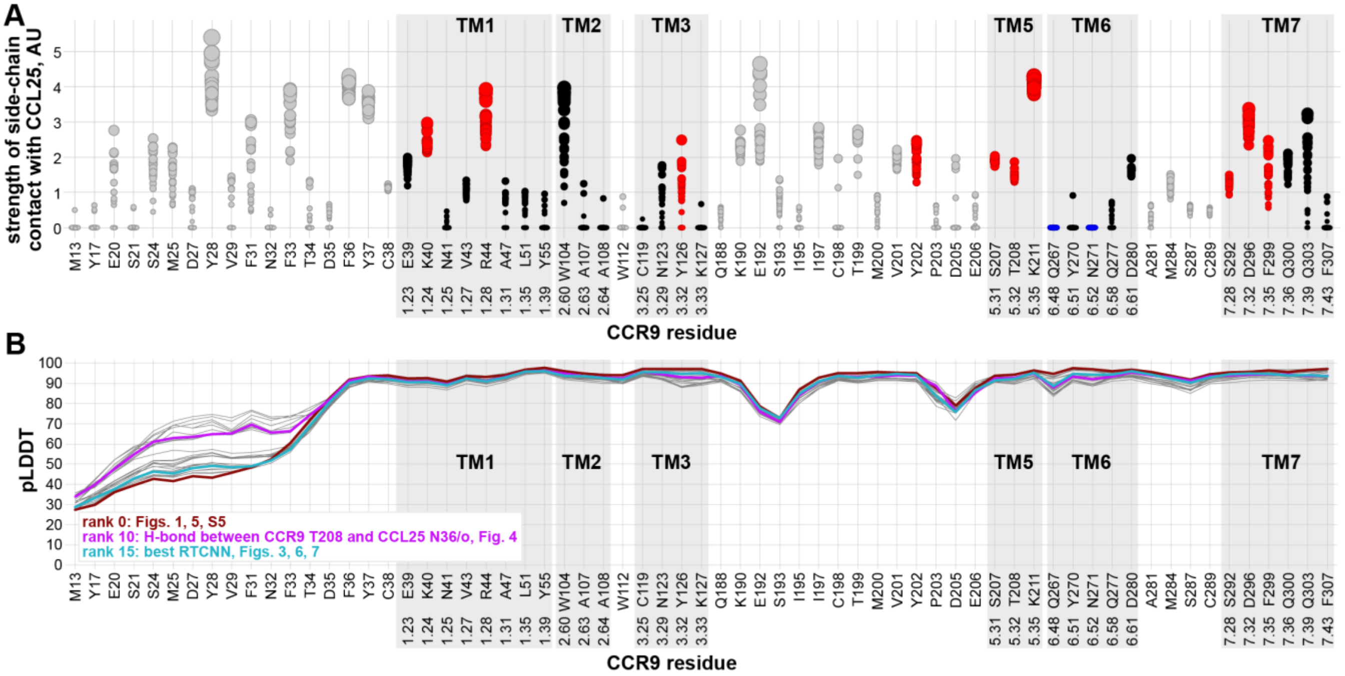

Supplementary Figure 6. Rationale for residue selection for mutagenesis.

(**A**) The y-axis, and the size of each circle, represent the contact that the sidechain of the corresponding residue makes with CCL25 in one of the models. Contact 'strengths' (an alternative representation of interatomic distances) were calculated as in the GPCR Dock assessments [1-3]. The data shown is an aggregation of 25 modes from the AF2 ensemble. Red columns denote CCR9 residues selected for mutagenesis based on chemokine contact analysis, blue are CCR9 residues Q267$^{6.48}$ and N271$^{6.52}$ that make no direct contacts with the chemokine but occupy positions known to mediate signal transmission in multiple Class A GPCRs. (**B**) The AF2-generated pLDDT scores for all CCR9 residues that contact CCL25 in any of the models in the ensemble, as well as for residues Q267$^{6.48}$ and N271$^{6.52}$ that were also selected for mutagenesis.

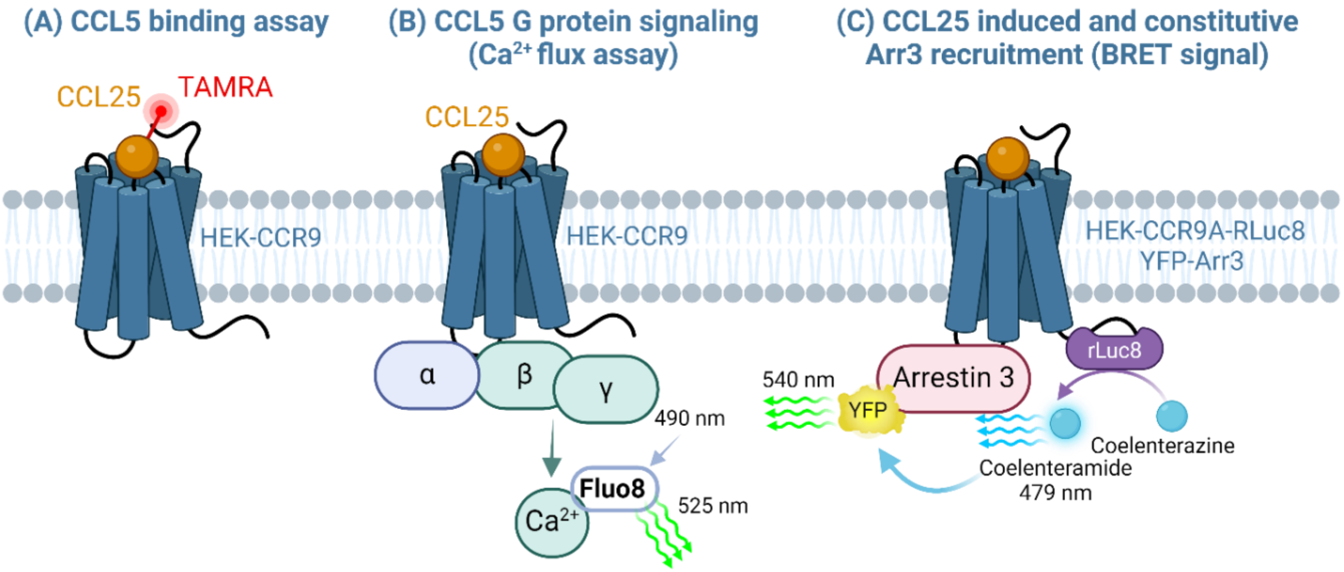

Supplementary Figure 7. Strategy for evaluating the roles of CCR9 residues in chemokine binding and signaling.

Evaluation of the impact that the 12 selected CCR9 CRS2 mutations have on CCR9 function, including their ability to bind CCL25 (**A**), to promote intracellular calcium (Ca2+) mobilization (**B**), and to recruit arrestin 3 in response to CCL25 (**C**). Figure created in https://BioRender.com.

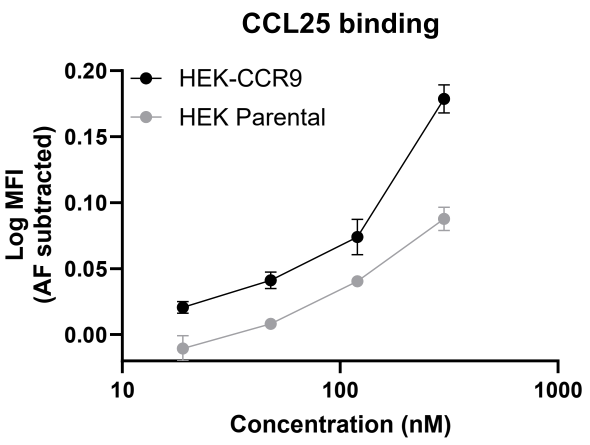

Supplementary Figure 8. Concentration-response curve for the binding of TAMRA-labelled CCL25 to HEK-CCR9 and HEK293 parental cell lines.

HEK293 parental cell line and HEK293 expressing WT CCR9 (HEK-CCR9) cells were incubated with TAMRA-labelled CCL25 at the indicated concentrations at 4°C for 1h. Binding signals are expressed as log MFI – log AF. Data represent mean ± SEM of triplicates in one experiment and are representative of 6 independent experiments.

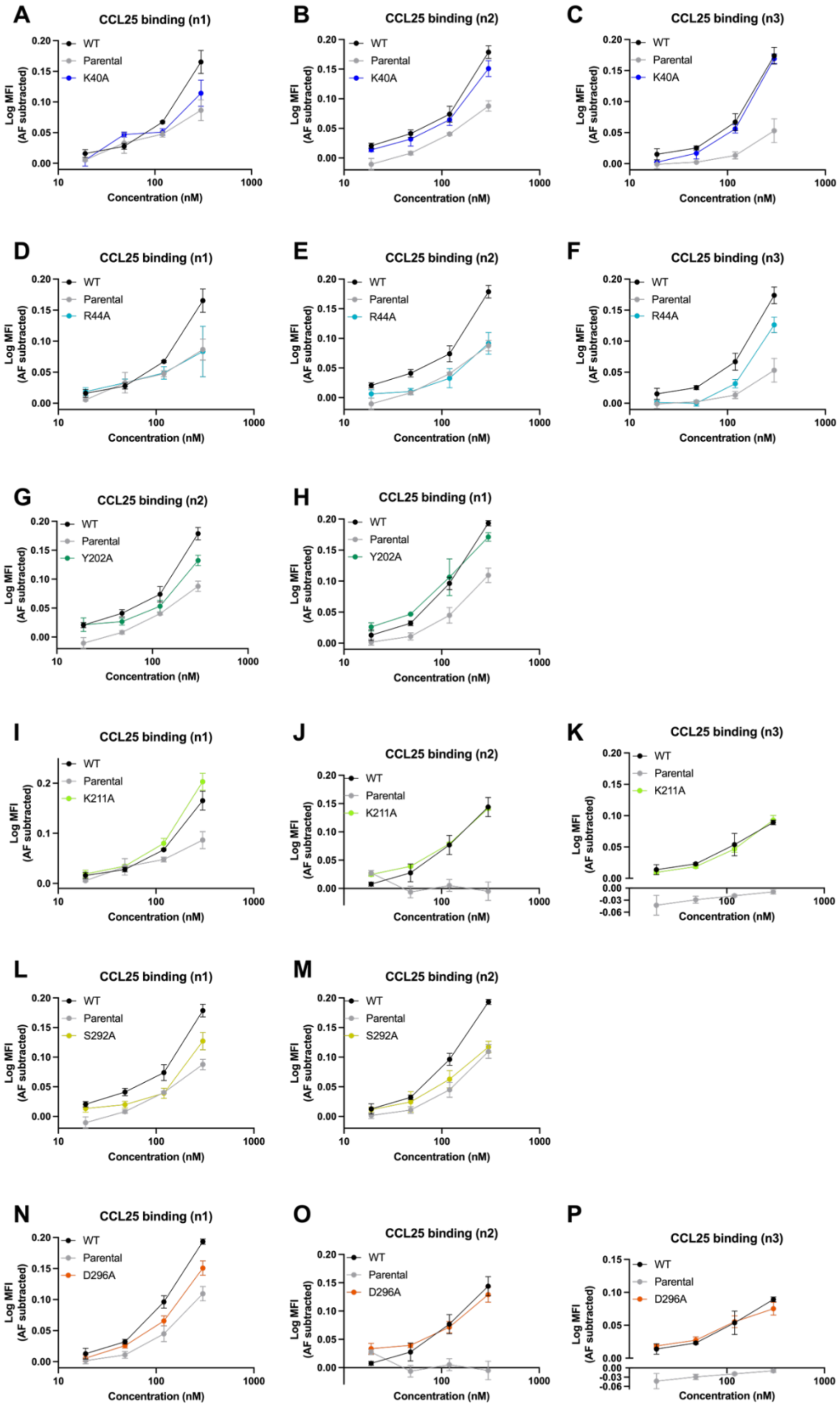
A
CCL25 binding (n1)
WT
Parental
K40A
B
CCL25 binding (n2)
WT
Parental
K40A
C
CCL25 binding (n3)
WT
Parental
K40A
D
CCL25 binding (n1)
WT
Parental
R44A
E
CCL25 binding (n2)
WT
Parental
R44A
F
CCL25 binding (n3)
WT
Parental
R44A
G
CCL25 binding (n2)
WT
Parental
Y202A
H
CCL25 binding (n1)
WT
Parental
Y202A
I
CCL25 binding (n1)
WT
Parental
K211A
J
CCL25 binding (n2)
WT
Parental
K211A
K
CCL25 binding (n3)
WT
Parental
K211A
L
CCL25 binding (n1)
WT
Parental
S292A
M
CCL25 binding (n2)
WT
Parental
S292A
N
CCL25 binding (n1)
WT
Parental
D296A
O
CCL25 binding (n2)
WT
Parental
D296A
P
CCL25 binding (n3)
WT
Parental
D296A
Log MFI (AF subtracted)
Concentration (nM)

Supplementary Figure 9. Independent experiments measuring CCL25 binding to CCR9 mutants from main-text Figure 3.

CCL25 concentration response binding curves are shown for CCR9 K40$^{1.24}$A (**A-C**), R44$^{1.28}$A (**D-F**), Y202^{ECL2}A (**G-H**), K211$^{5.35}$A (**I-K**), S292$^{7.28}$A (**L-M**) and D296$^{7.32}$A (**N-P**) compared to CCR9 WT and parental cells measured in the same experiments. To obtain the curves, HEK293 parental cell line (parental) and HEK293 cells expressing WT or mutant CCR9 were incubated at 4°C for 1h with TAMRA-labelled chemokine CCL25 at serial dilutions (300, 120, 48, 19 nM). Binding signals are expressed as log MFI – log AF where AF (autofluorescence) is the MFI of the corresponding cell line in the same experiment in the absence of the fluorescent chemokine. Data points represent mean ± SEM of triplicates in one experiment.

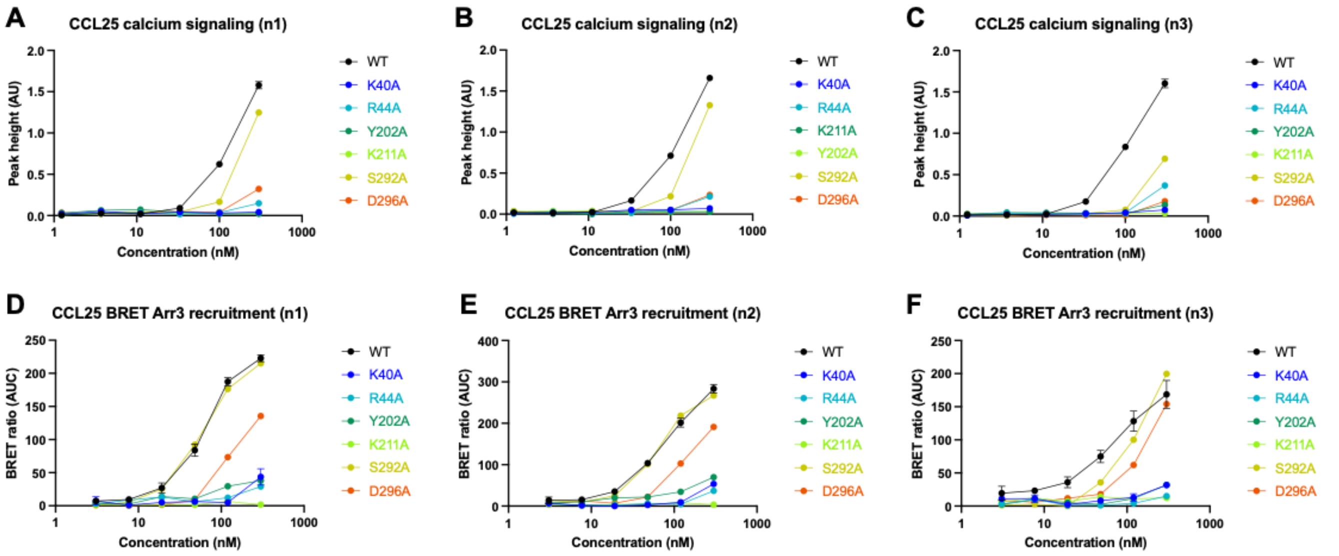

Supplementary Figure 10. Independent experiments measuring CCL25-induced Ca^{2+} flux and BRET Arr3 recruitment to CCR9 mutants from main-text Figure 3.

CCL25 concentration-response curves for the independent experiments measuring Ca^{2+} flux response (**A-C**) and BRET Arr3 recruitment (**D-F**) in K40$^{1.24}$A, R44$^{1.28}$A, Y202^{ECL2}A, K211$^{5.35}$A, S292$^{7.28}$A and D296$^{7.32}$A CCR9 mutants, compared to CCR9 WT measured in the same experiment. For each mutant, 3 independent experiments were performed and are shown. **(A-C)** CCL25-induced Ca^{2+} signaling on WT CCR9 or CCR9 mutants expressed in HEK293 cells. Ca^{2+} signals in response to CCL25 at the indicated concentrations are shown as mean peak height (**eq. 3**) ± SEM from triplicate wells. **(D-F)** BRET assays for CCL25-induced Arr3 recruitment on WT CCR9 and CCR9 mutants. Data points represent mean ± SEM of normalized BRET signal AUCs (**eq. 4**) obtained in triplicate wells.

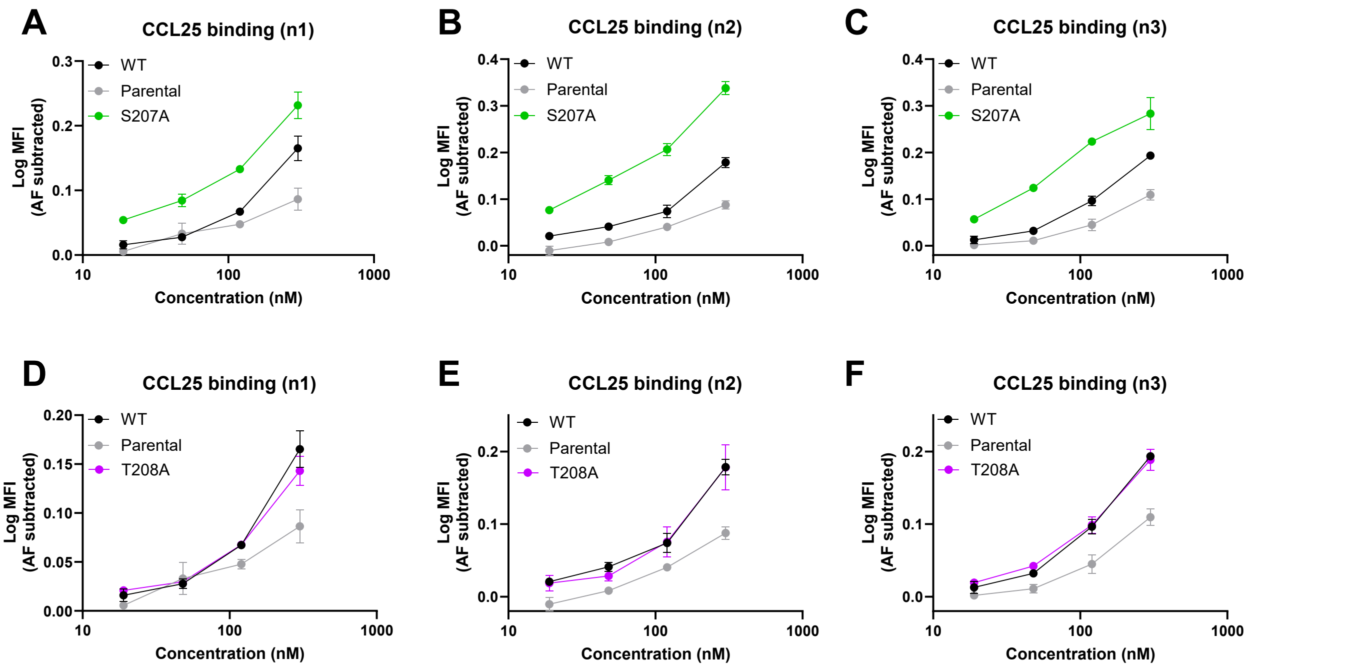

Supplementary Figure 11. Independent experiments measuring CCL25 binding to CCR9 mutants from main-text Figure 4.

CCL25 concentration response binding curves are shown for CCR9 S207$^{5.31}$A (**A-C**) and T208$^{5.32}$A (**D-F**) compared to CCR9 WT and parental cells measured in the same experiments. Refer to **Supplementary Fig. 9** legend for details.

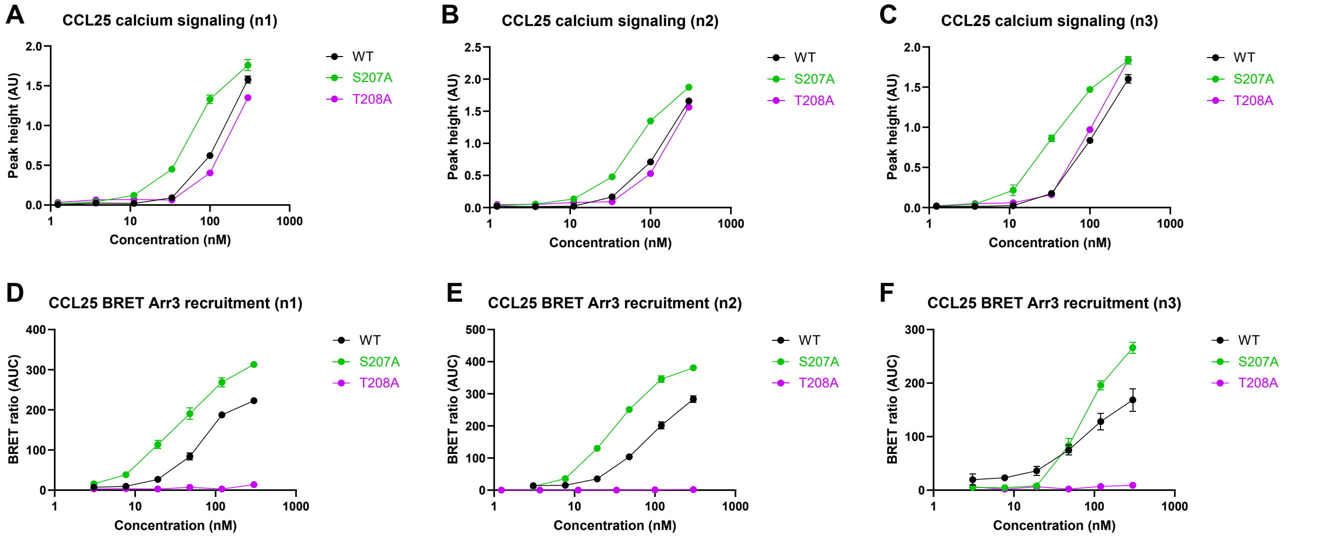

Supplementary Figure 12. Independent experiments measuring CCL25-induced Ca2+ flux and BRET Arr3 recruitment to CCR9 mutants from main-text Figure 4.

CCL25 concentration-response curves for the independent experiments measuring Ca^{2+} flux response (**A-C**) and BRET Arr3 recruitment (**D-F**) in S207$^{5.31}$A and T208$^{5.32}$A CCR9 mutants compared to CCR9 WT measured in the same experiment. For each mutant, 3 independent experiments were performed and are shown. Refer to **Supplementary Fig. 10** legend for details**.**

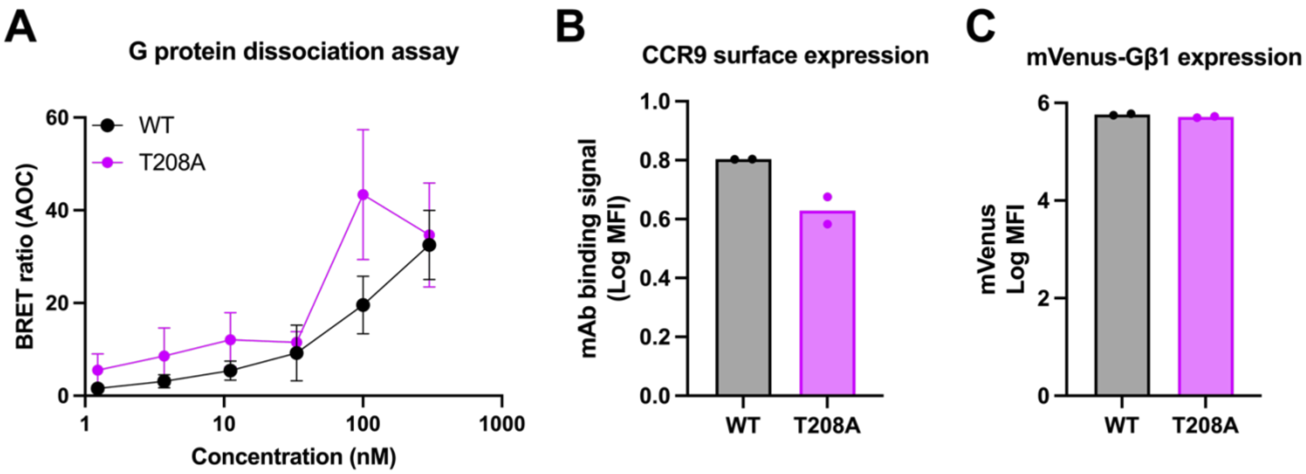

Supplementary Figure 13. G protein dissociation BRET assay on HEK-CCR9-T208$^{5.32}$A.

(A) HEK293 cells expressing WT or mutant CCR9 (T208$^{5.32}$A) co-transfected with Gαi(91)-Rluc2, mVenus-Gβ1 and Gγ2 were stimulated with CCL25 at the indicated concentrations and BRET signals were measured. Data points represent mean ± SEM of area over the curve (AOC) of the BRET (eq. 4) obtained in technical triplicates in one experiment and are representative of 2 independent experiments. **(B)** HEK293 cells expressing WT or mutant CCR9 (T208$^{5.32}$A) were incubated with anti-CCR9 mAb at 4°C for 1h and analysed by flow cytometry. Signals are expressed as $\log MFI_{\text{WT or T208A}} - \log MFI_{\text{parental}}$. Data represent mean log MFI ± SEM from two independent experiments. **(C)** Transfection efficiency of mVenus-Gβ1 was evaluated by measuring mVenus fluorescence by flow cytometry. Signals are expressed as $\log MFI_{\text{WT or T208A}} - \log MFI_{\text{parental}}$. Data represent mean log MFI ± SEM from two independent experiments.

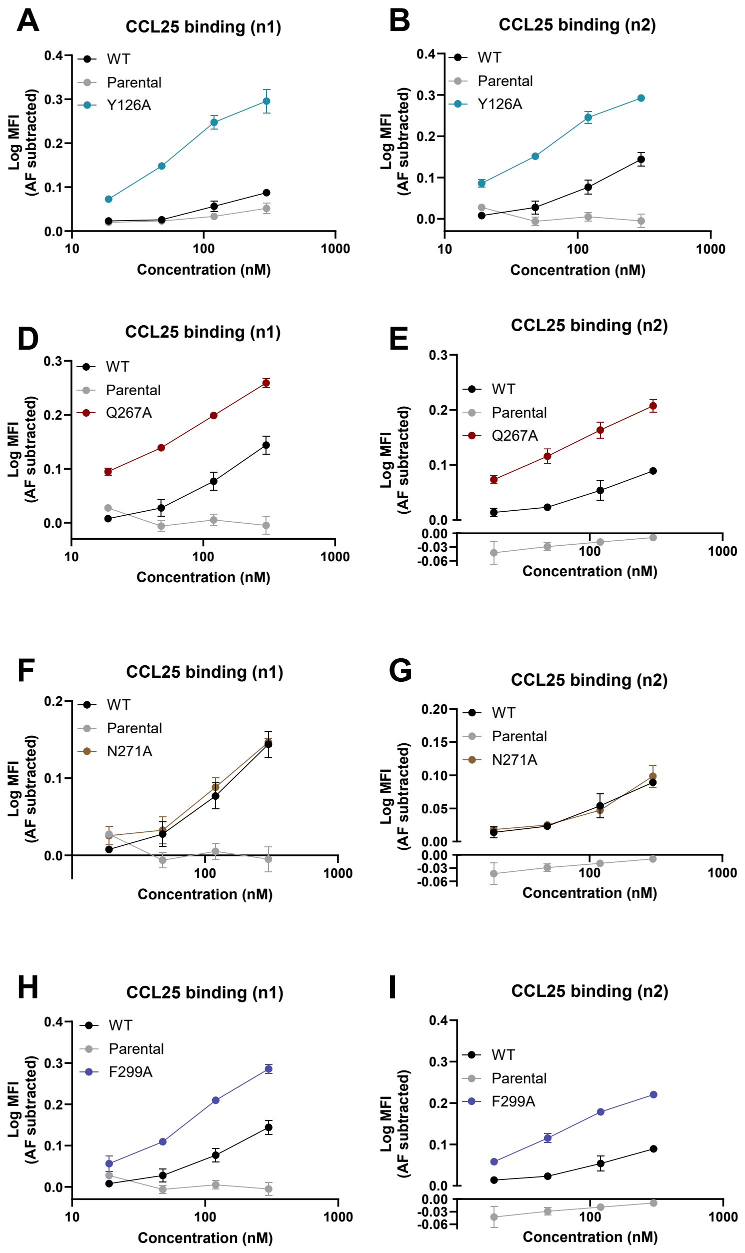

Supplementary Figure 14. Independent experiments measuring CCL25 binding to CCR9 mutants from main-text Figure 5.

CCL25 concentration response binding curves are shown for CCR9 Y126$^{3.32}$A (**A-C**), Q267$^{6.48}$A (**D-F**), N271$^{6.52}$A (**G-I**), and F299$^{7.35}$A (**J-L**) compared to CCR9 WT and parental cells measured in the same experiments. Refer to **Supplementary Fig. 9** legend for details.

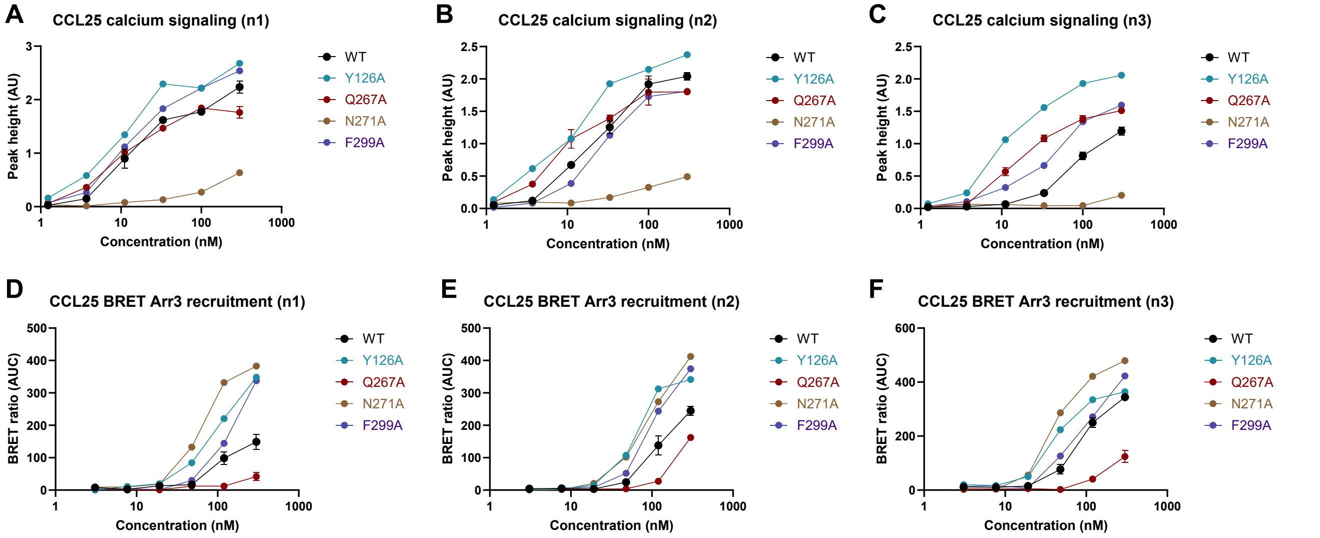

Supplementary Figure 15. Independent experiments measuring CCL25-induced Ca^{2+} flux and BRET Arr3 recruitment to CCR9 mutants from main-text Figure 5.
CCL25 concentration-response curves for the independent experiments measuring Ca^{2+} flux response (**A-C**) and BRET Arr3 recruitment (**D-F**) in Y126$^{3.32}$A, Q267$^{6.48}$A, N271$^{6.52}$A, and F299$^{7.35}$A CCR9 mutants compared to CCR9 WT measured in the same experiment. For each mutant, 3 independent experiments were performed and are shown. Refer to **Supplementary Fig. 10** legend for details**.**

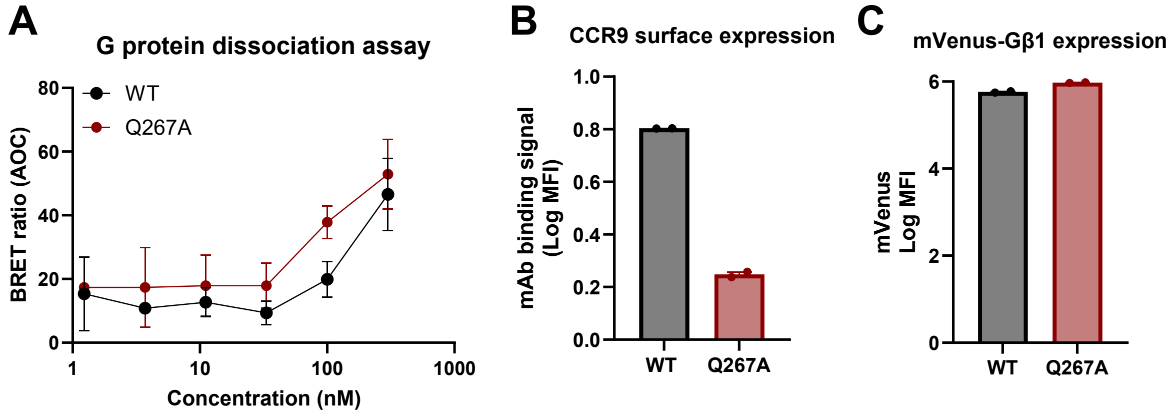

Supplementary Figure 16. G protein dissociation BRET assay on HEK-CCR9-Q267$^{6.48}$A.
Refer to **Supplementary Fig. 13** legend for details.

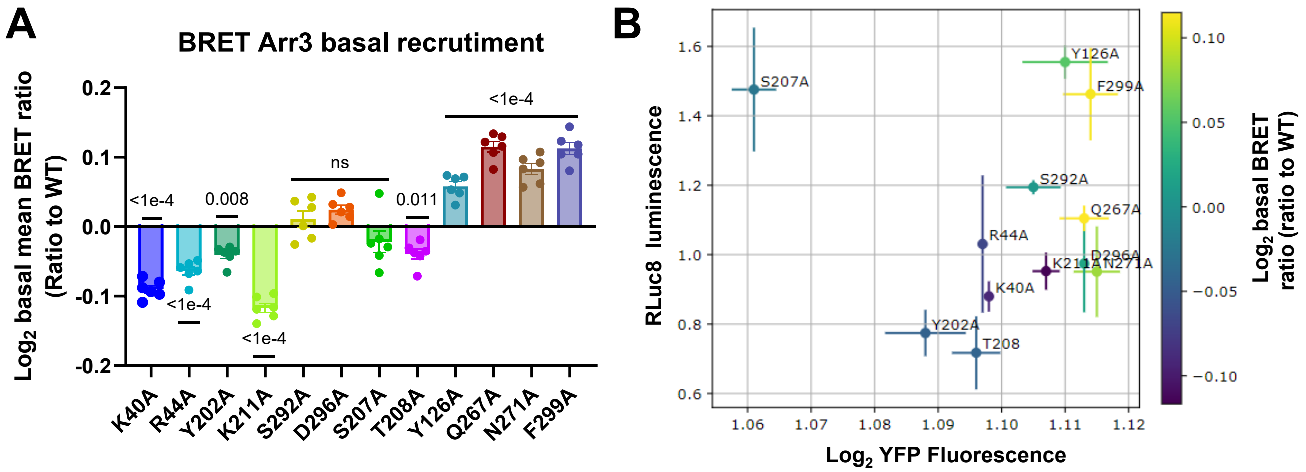

Supplementary Figure 17. Characterization of HEK-CCR9-RLuc8 cell lines with respect to basal arrestin recruitment and acceptor:donor ratios.

A. HEK293 cells stably co-expressing YFP-Arr3 and WT or mutant CCR9-RLuc8 were incubated with BRET buffer containing 5 µM coelenterazine h and basal BRET signals were measured for 7 min. Data points represent the mean ± SEM of log_2 transformed BRET signal normalized to WT receptor of 6 independent experiments. P-values were calculated using one-way ANOVA with post-hoc tests and Holm-Šídák's correction for multiple comparisons on log-transformed mutant-to-WT ratios. **B.** Scatter plot of basal Arr3-YFP fluorescence vs basal CCR9-RLuc8 luminescence, both relative to WT cell line measured in the same experiment (log_2 transformed axis). Plot points are colored according to basal BRET expressed as in panel **A**. Points represent mean ± SEM of RLuc8 luminescence (y axis) and YFP fluorescence (x axis) relative to WT receptor of 2 independent experiments.

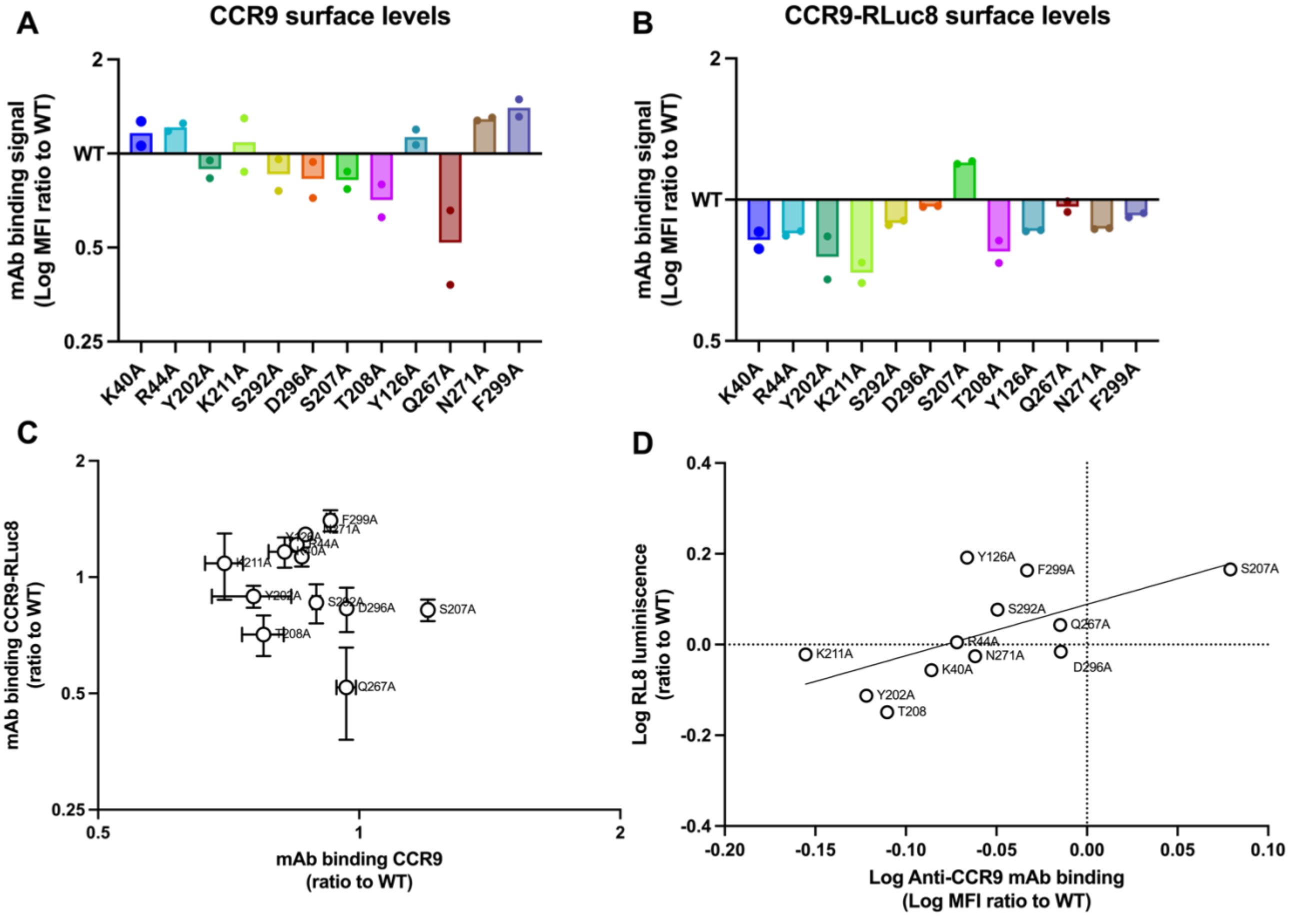

Supplementary Figure 18. Characterization of HEK-CCR9 and HEK-CCR9-RLuc8 YFP-Arr3 cell lines.
(A-B) HEK293 cells expressing WT or mutant CCR9 (**A**) and HEK293 cells stably co-expressing YFP-Arr3 and WT or mutant CCR9-RLuc8 (**B**) were incubated with anti-CCR9 mAb at 4°C for 1h and analysed by flow cytometry. Signals are expressed as ratios to WT (eq. 1). **(C)** Comparison between mAb binding to HEK-CCR9 untagged and HEK-CCR9-RLuc8 mutants shows no correlation indicating the lack of antibody sensitivity to the individual mutations. **(D)** Comparison between luminescence and anti-CCR9 mAb binding, relative to WT receptor, for CCR9-RLuc8 mutants. Data points represent mean of luminescence (y axis) and mAb binding (x axis) relative to WT receptor of 2 independent experiments (simple linear regression: R^2 = 0.3940, p-value = 0.0289).

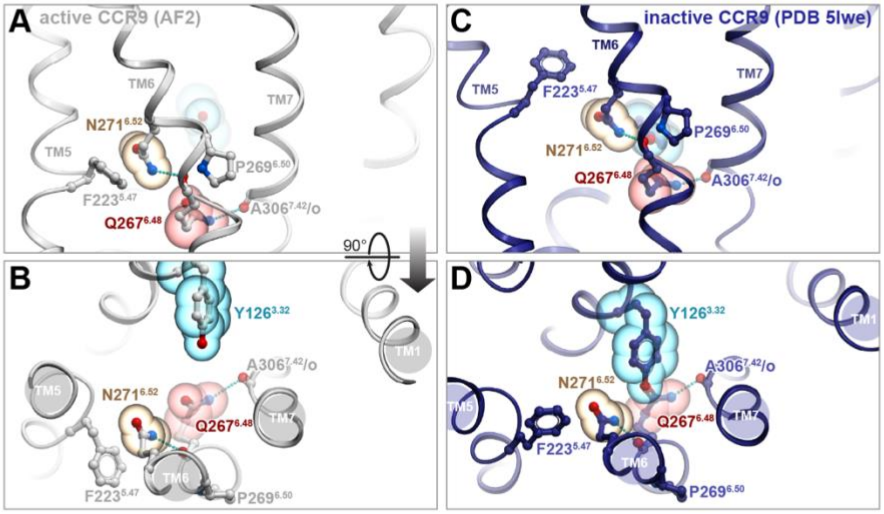

Supplementary Figure 19. Pocket floor residues Q267$^{6.48}$ and N271$^{6.52}$ interlock CCR9 TM6 to TM7 and TM5, respectively.

N271$^{6.52}$ pi-stacks with F223$^{5.47}$ of TM5, whereas Q267$^{6.48}$ hydrogen-bonds to the backbone oxygen of A306$^{7.42}$ of TM7. (**A-B)** active-state AF2 model; (**C-D**) inactive antagonist-bound structure, PDB entry 5LWE. In (**A**) and (**C**), the base of the binding pocket is viewed parallel to the membrane; in (**B**) and (**D**), the same region is viewed perpendicular to membrane from the extracellular side.

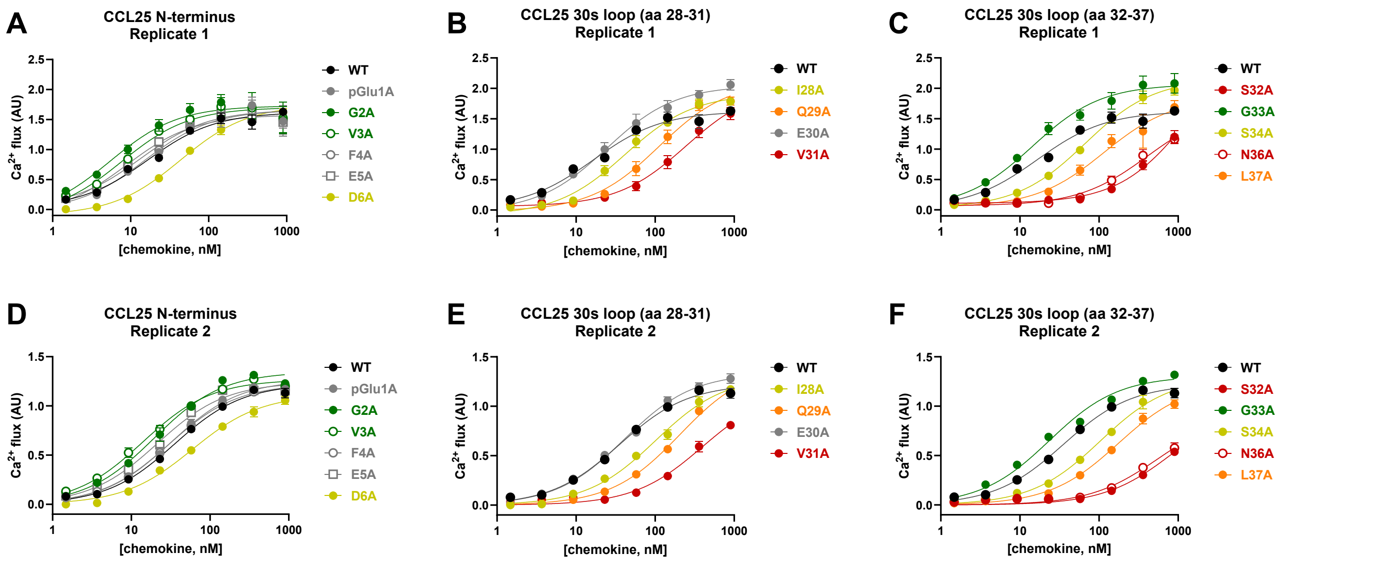

Supplementary Figure 20. Concentration-dependent responses of MOLT-4 cells to WT CCL25 and the indicated CCL25 mutants in the independent experiments measuring intracellular Ca^{2+} mobilization. Data points represent mean ± SD for peak height obtained in technical triplicates in 2 independent experiments (experiment 1 (**A-C**) and experiment 2 (**D-F**)).

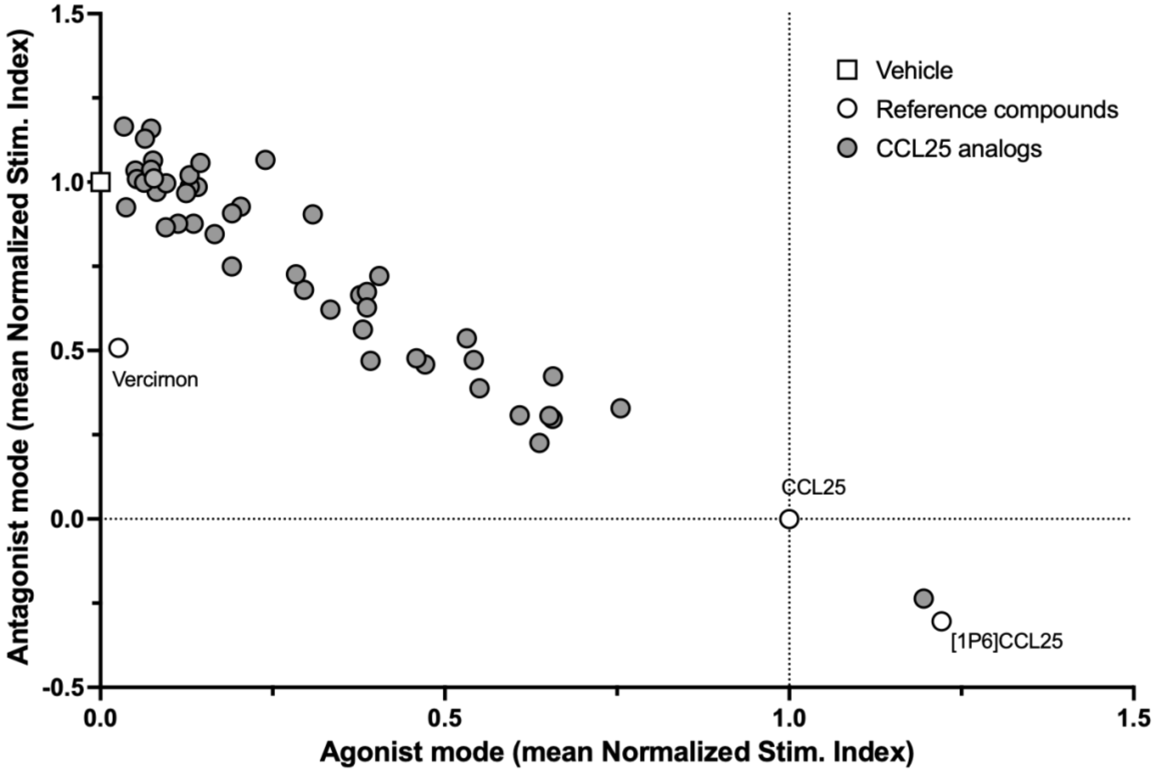

Supplementary Figure 21. Activity of the CCL25 N-terminally extended and natural-length analogs discovered by phage display.

Agonist mode stimulation indexes (SIs) correspond to AUCRC of Ca^{2+} flux responses of MOLT-4 cells to CCL25 analogs. Antagonist mode stimulation indexes (SIs) correspond to AUCRC upon subsequent treatment with CCL25 (100 nM). Vercirnon was used as a positive control antagonist. SIs were normalized by the AUCRC of WT CCL25 (stimulation index set to 1 for agonist mode and to 0 for antagonist mode) and by the AUCRC of vehicle + 100 nM CCL25 (SI set to 0 for agonist mode and to 1 for antagonist mode).

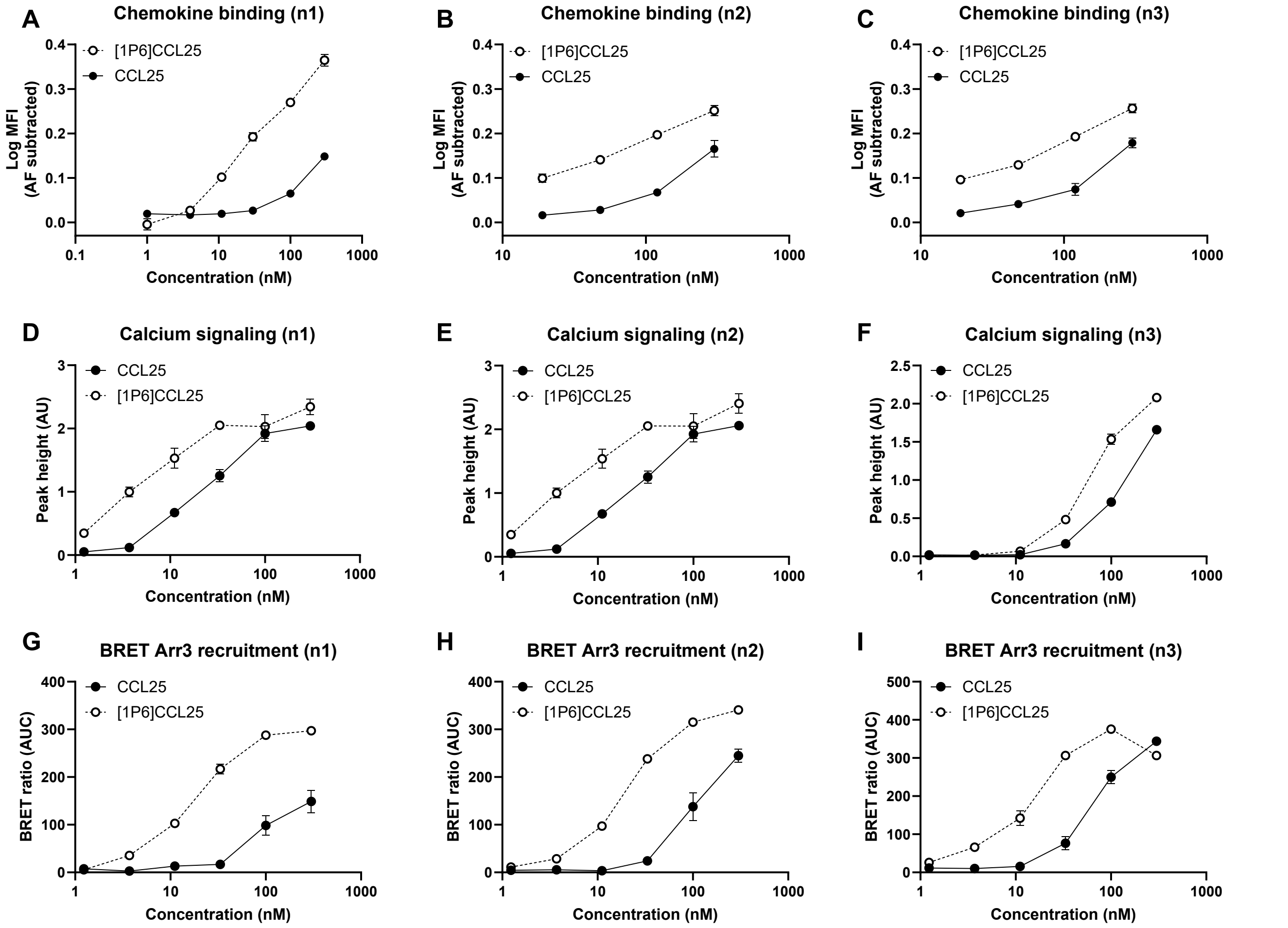

Supplementary Figure 22. Concentration-response curves for the independent experiments measuring [1P6]CCL25 binding, Ca^{2+} flux and BRET Arr3 recruitment to WT CCR9 stably expressed in HEK293 cells. **(A-C)** CCL25 and [1P6]CCL25 binding to CCR9 stably expressed in HEK293 cells. HEK293 cells stably expressing CCR9 were incubated at 4°C for 1h with the indicated concentrations of TAMRA-labelled CCL25 or [1P6]CCL25. Signals are expressed as Log $MFI_{chemokine}$ – Log MFI_{AF}. Data represent mean ± SEM of binding signal from triplicate wells within a single experiment; 3 independent experiments were performed and are shown. **(D-F)** CCL25 and [1P6]CCL25-induced Ca^{2+} signaling on WT CCR9 expressed in HEK293 cells. Ca^{2+} signals in response to CCL25 and [1P6]CCL25 at the indicated concentrations are shown as mean peak height (**eq. 3**) ± SEM from triplicate wells within a single experiment; 3 independent experiments were performed and are shown. (**G-I)** BRET assays for CCL25 and [1P6]-CCL25-induced Arr3 recruitment on WT CCR9. Data points represent mean ± SEM of BRET signal (**eq. 4**) obtained in triplicate wells within a single experiment; 3 independent experiments were performed and are shown.

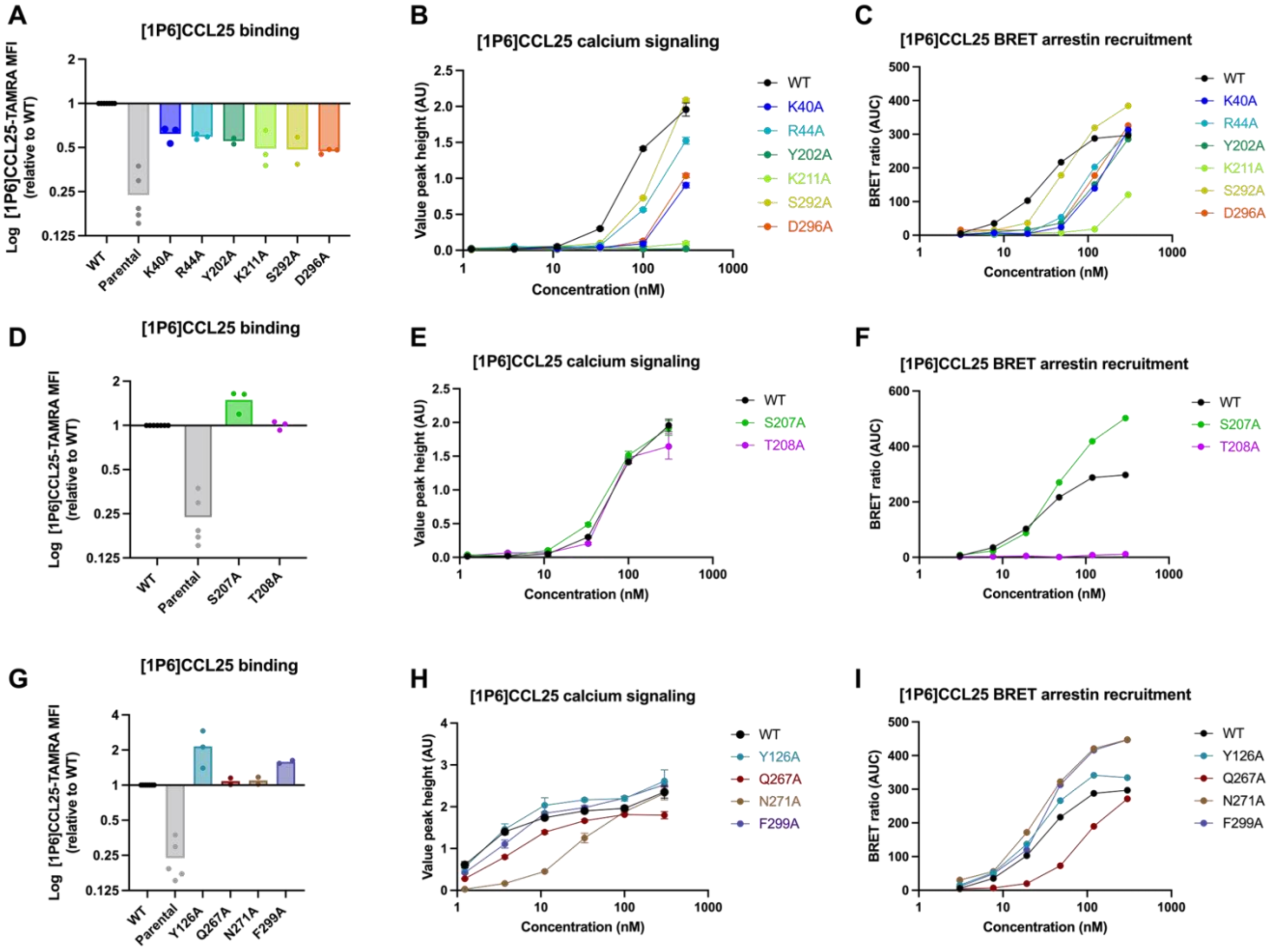

Supplementary Figure 23. Binding of [1P6]CCL25 and concentration-response curves for [1P6]CCL25-induced Ca^{2+} flux and BRET Arr3 recruitment to WT and mutant CCR9 stably expressed in HEK293 cells. Statistical analyses are shown in **Supplementary Table 4. (A, D, G)** [1P6]CCL25 binding to WT CCR9 and CCR9 mutants expressed in HEK293 cells. Cells were incubated at 4°C for 1h with 300 nM of TAMRA-labelled [1P6]CCL25. Bars represent mean ± SEM of the ratio of specific binding signals (**eq. 2**) between the mutant and WT CCR9, measured in 2-3 independent experiments. P-values (one way ANOVA with post-hoc tests and Holm-Šídák's correction for multiple comparisons on log-transformed binding ratios, eq. 2) in comparison to HEK-CCR9 WT and HEK293 parental cells are shown for each mutant in black and grey, respectively. Complete CCL25 binding CRCs are available in **Supplementary Figs. 24, 25 and 26**. **(B, E, H)** [1P6]CCL25-induced Ca^{2+} signaling on WT CCR9 or CCR9 mutants expressed in HEK293 cells. Ca^{2+} signals in response to [1P6]CCL25 at the indicated concentrations are shown as mean peak height (**eq. 3**) ± SEM from triplicate wells; the data shown are representative of 3 independent experiments (complete data set is available in **Supplementary Figs. 27, 28 and 29**). (**C, F, I)** BRET assays for [1P6]CCL25-induced Arr3 recruitment on WT CCR9 and CCR9 mutants. Data points represent mean ± SEM of BRET signal (**eq. 4**) obtained in triplicate wells; data shown are representative of 3 independent experiments (complete data set is available in **Supplementary Figs. 27, 28 and 29**).

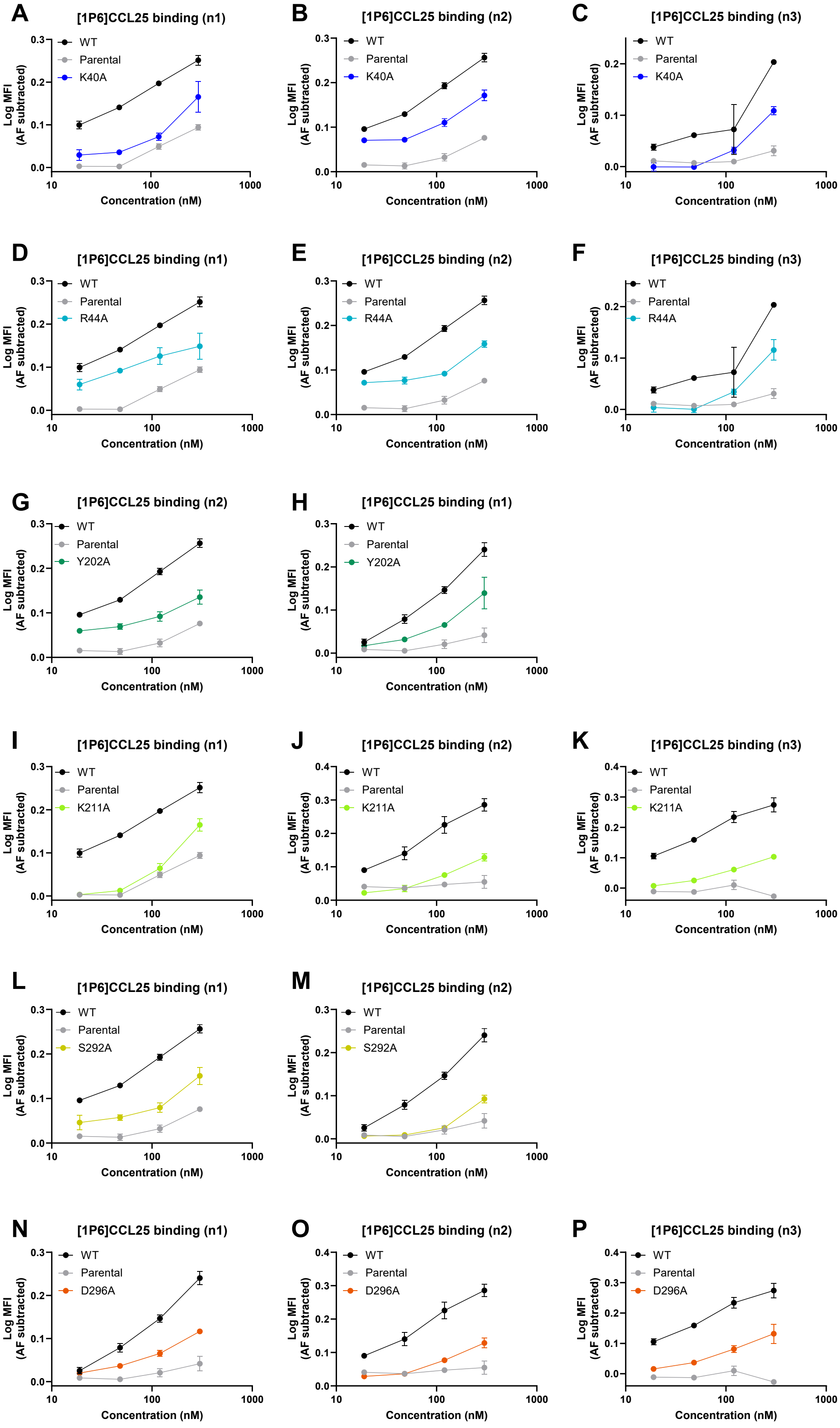
A
[1P6]CCL25 binding (n1)
WT
Parental
K40A
B
[1P6]CCL25 binding (n2)
WT
Parental
K40A
C
[1P6]CCL25 binding (n3)
WT
Parental
K40A
D
[1P6]CCL25 binding (n1)
WT
Parental
R44A
E
[1P6]CCL25 binding (n2)
WT
Parental
R44A
F
[1P6]CCL25 binding (n3)
WT
Parental
R44A
G
[1P6]CCL25 binding (n2)
WT
Parental
Y202A
H
[1P6]CCL25 binding (n1)
WT
Parental
Y202A
I
[1P6]CCL25 binding (n1)
WT
Parental
K211A
J
[1P6]CCL25 binding (n2)
WT
Parental
K211A
K
[1P6]CCL25 binding (n3)
WT
Parental
K211A
L
[1P6]CCL25 binding (n1)
WT
Parental
S292A
M
[1P6]CCL25 binding (n2)
WT
Parental
S292A
N
[1P6]CCL25 binding (n1)
WT
Parental
D296A
O
[1P6]CCL25 binding (n2)
WT
Parental
D296A
P
[1P6]CCL25 binding (n3)
WT
Parental
D296A
Log MFI (AF subtracted)
Concentration (nM)
10
100
1000

Supplementary Figure 24. Independent experiments measuring [1P6]CCL25 binding to CCR9 mutants from Supplementary Fig. 23A.

[1P6]CCL25 concentration response binding curves are shown for CCR9 $K40^{1.24}A$ (**A-C**), $R44^{1.28}A$ (**D-F**), $Y202^{ECL2}A$ (**G-H**), $K211^{5.35}A$ (**I-K**), $S292^{7.28}A$ (**L-M**) and $D296^{7.32}A$ (**N-P**) compared to CCR9 WT and parental cells measured in the same experiments. Refer to **Supplementary Fig. 9** legend for details.

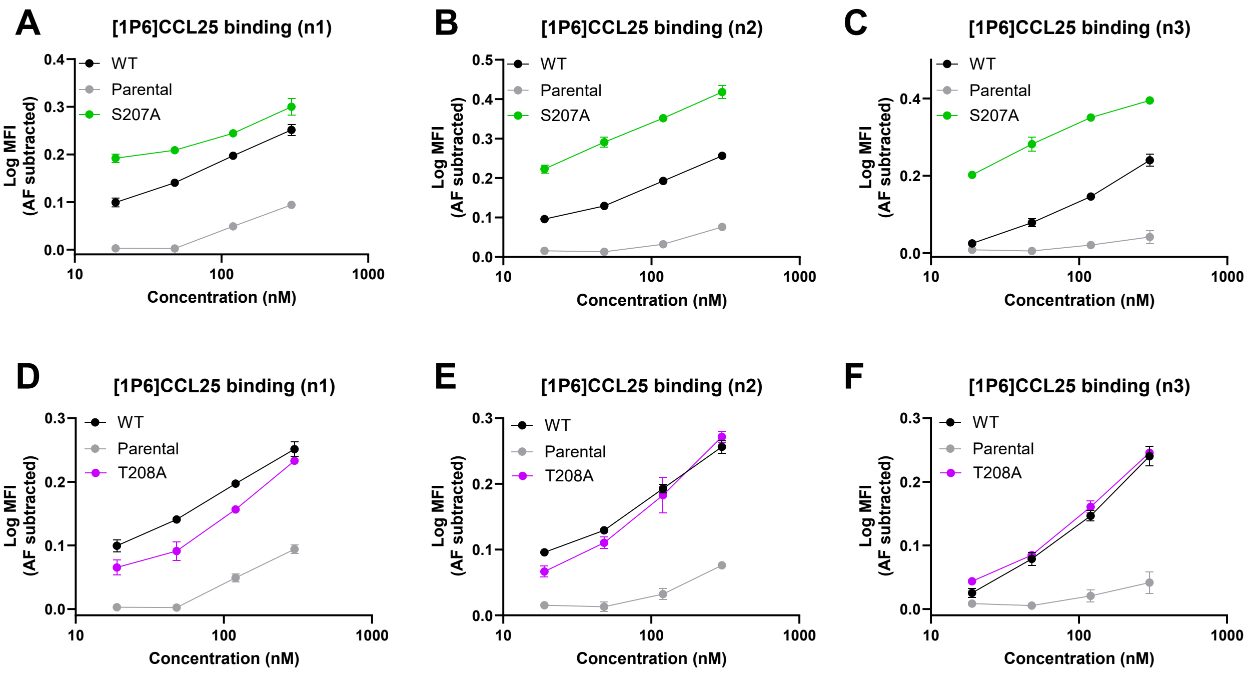

Supplementary Figure 25. Independent experiments measuring [1P6] CCL25 binding to CCR9 mutants from Supplementary Fig. 23D.

[1P6]CCL25 concentration response binding curves are shown for CCR9 S207$^{5.31}$A (**A-C**) and T208$^{5.32}$A (**D-F**) compared to CCR9 WT and parental cells measured in the same experiments. Refer to **Supplementary Fig. 9** legend for details.

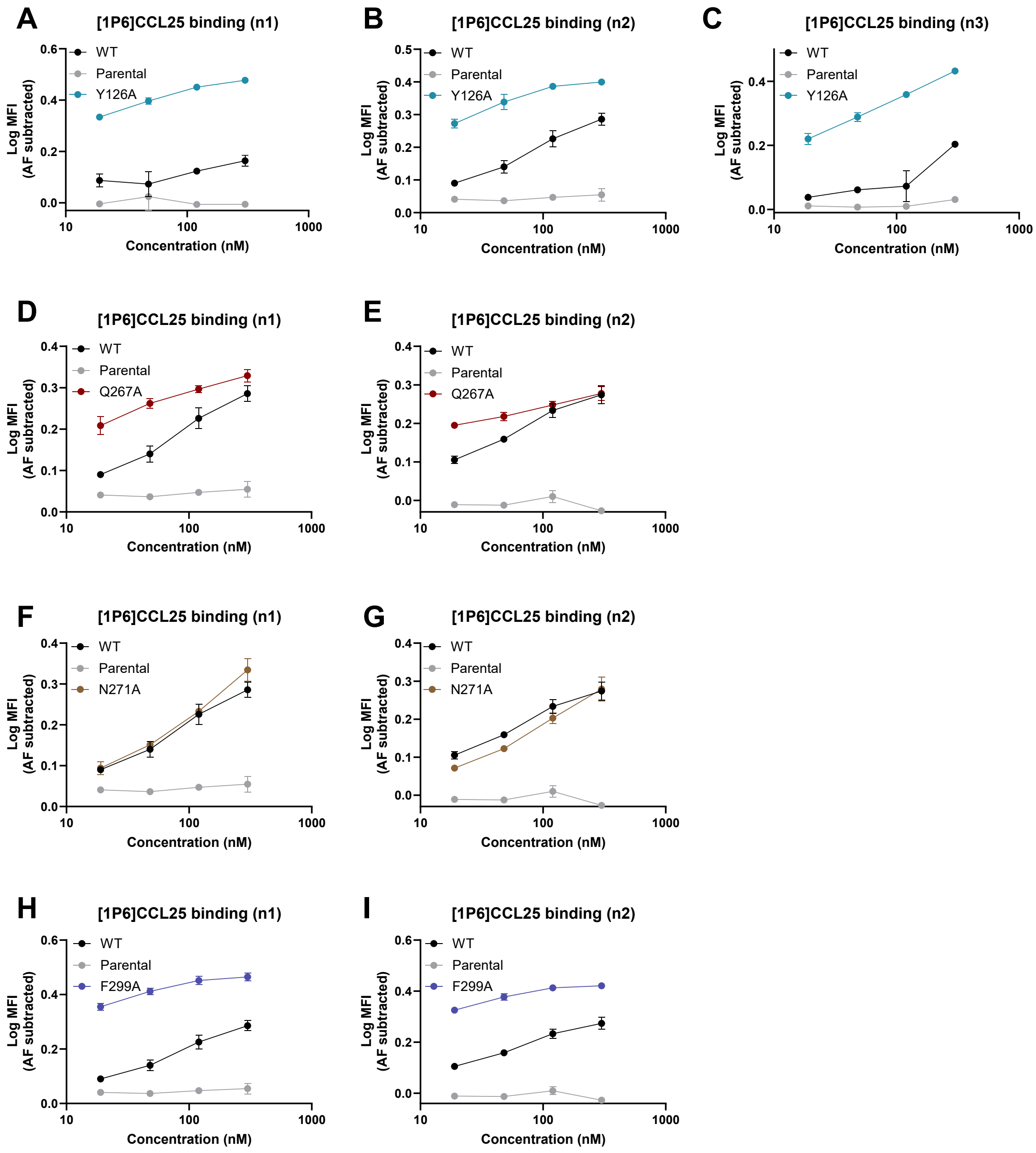

Supplementary Figure 26. Independent experiments measuring [1P6]CCL25 binding to CCR9 mutants from Supplementary Fig. 23G.

[1P6]CCL25 concentration response binding curves are shown for CCR9 Y126$^{3.32}$A (**A-C**), Q267$^{6.48}$A (**D-F**), N271$^{6.52}$A (**G-I**), and F299$^{7.35}$A (**J-L**) compared to CCR9 WT and parental cells measured in the same experiments. Refer to **Supplementary Fig. 9** legend for details.

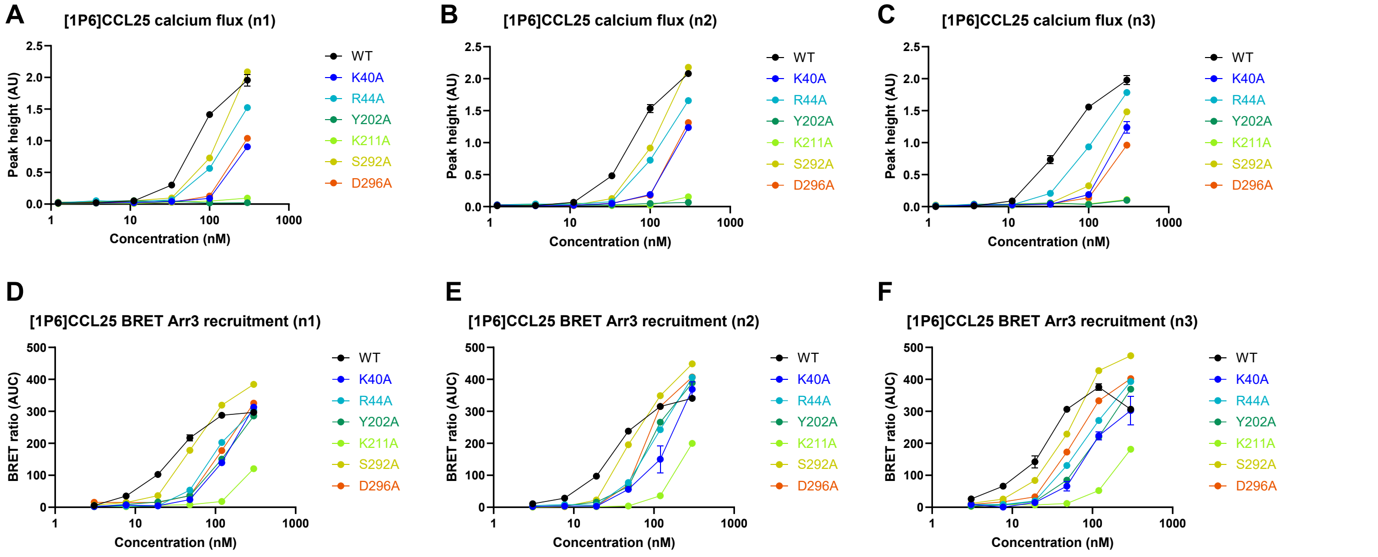

Supplementary Figure 27. Independent experiments measuring [1P6]CCL25-induced Ca^{2+} flux and BRET Arr3 recruitment to CCR9 mutants from Supplementary Figs. 23B and C.

[1P6]CCL25 concentration-response curves for the independent experiments measuring Ca^{2+} flux response (**A-C**) and BRET Arr3 recruitment (**D-F**) for CCR9 K40$^{1.24}$A, R44$^{1.28}$A, Y202^{ECL2}A, and K211$^{5.35}$A, S292$^{7.28}$A and D296$^{7.32}$A, compared to CCR9 WT measured in the same experiment. For each mutant, 3 independent experiments were performed and are shown. Refer to **Supplementary Fig. 10** legend for details.

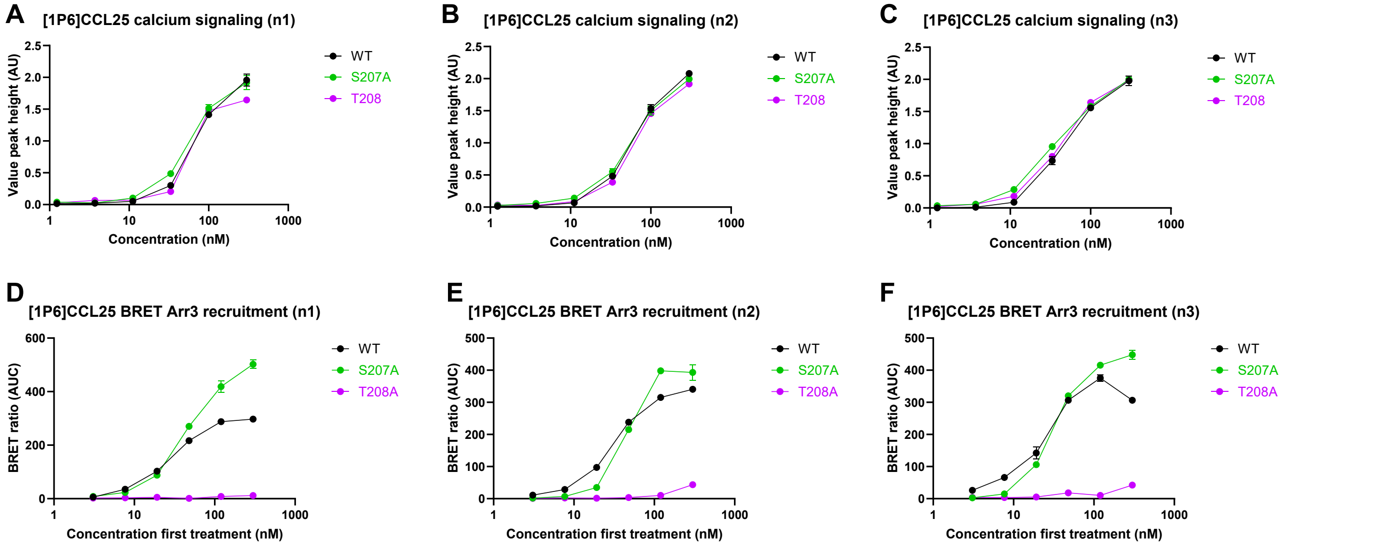

Supplementary Figure 28. Independent experiments measuring [1P6]CCL25-induced Ca^{2+} flux and BRET Arr3 recruitment to CCR9 mutants from Supplementary Figs. 23E and F.

[1P6]CCL25 concentration-response curves for the independent experiments measuring Ca^{2+} flux response (**A-C**) and BRET Arr3 recruitment (**D-F**) for CCR9 S207$^{5.31}$A and T208$^{5.32}$A, compared to CCR9 WT measured in the same experiment. For each mutant, 3 independent experiments were performed and are shown. Refer to **Supplementary Fig. 10** legend for details.

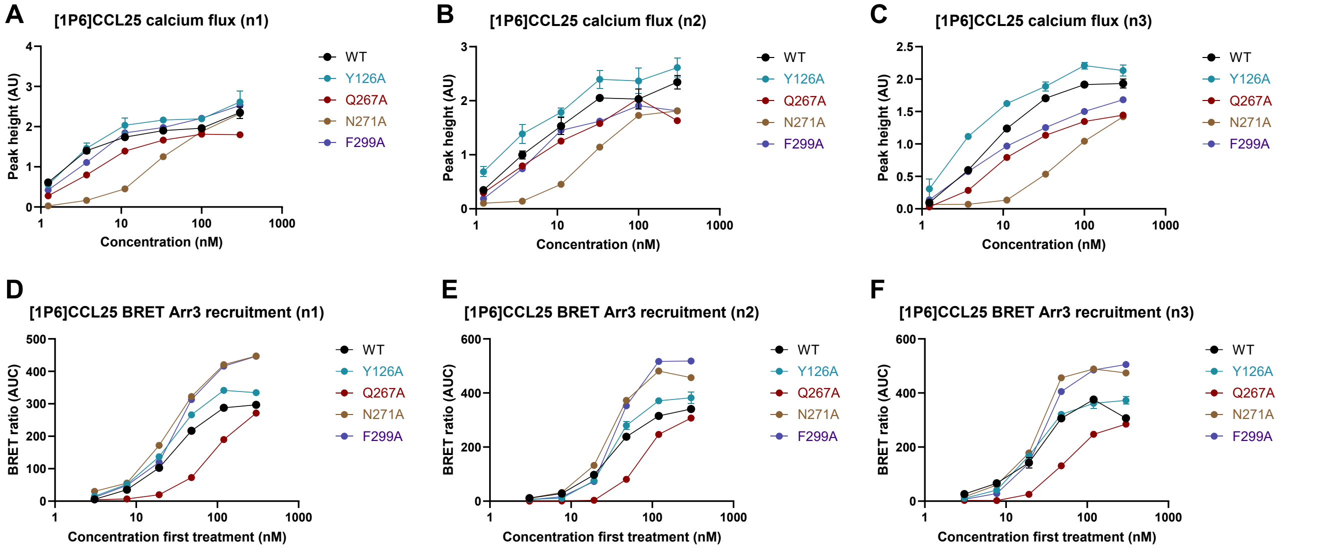

Supplementary Figure 29. Independent experiments measuring [1P6]CCL25-induced Ca^{2+} flux and BRET Arr3 recruitment to CCR9 mutants from Supplementary Fig. 23H and I.

[1P6]CCL25 concentration-response curves for the independent experiments measuring Ca^{2+} flux response (**A-C**) and BRET Arr3 recruitment (**D-F**) for CCR9 Y126$^{3.32}$A, Q267$^{6.48}$A, N271$^{6.52}$A, and F299$^{7.35}$A, compared to CCR9 WT measured in the same experiment. For each mutant, 3 independent experiments were performed and are shown. Refer to **Supplementary Fig. 10** legend for details.

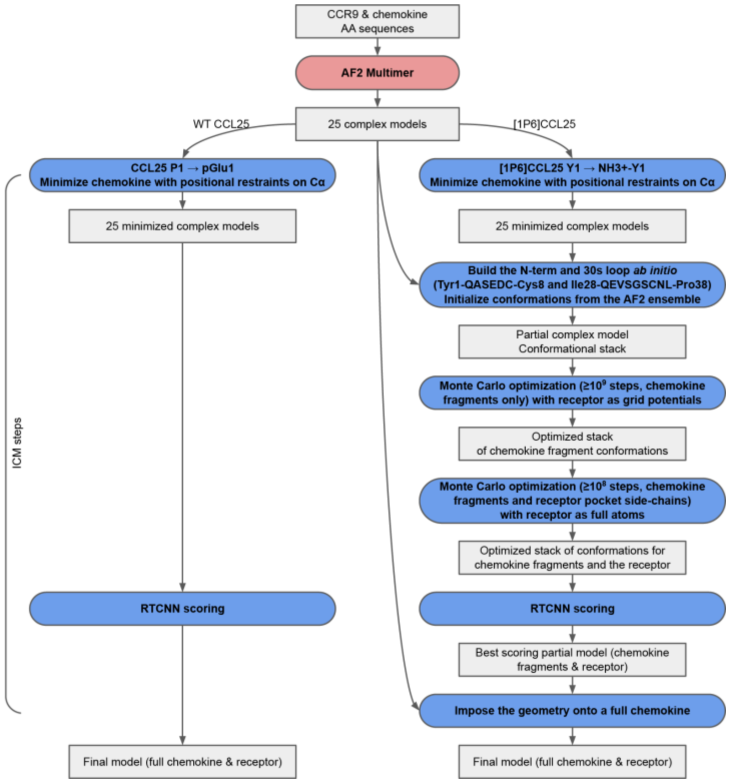

Supplementary Figure 30. Schematic overview of the CCR9 modeling workflow.
This figure illustrates the modeling strategy described in the Methods section "Model building".

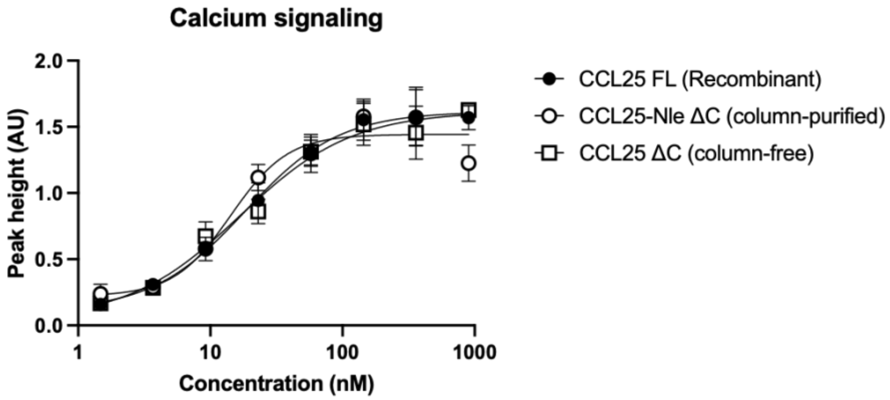

Supplementary Figure 31. Chemically synthesized C-terminally truncated CCL25 retains signaling potency and efficacy of full-length recombinant CCL25, with and without column purification.
Full length (FL) recombinant CCL25 and chemically synthesized C-terminally truncated CCL25 (ΔC) subjected or not to column purification (column-purified or column-free, respectively) were added to MOLT-4 cells at the indicated concentrations and Ca^{2+} flux signals were measured. Data points represent peak height (eq. 3) ± SEM from triplicate wells; the data shown are representative of 2 independent experiments.

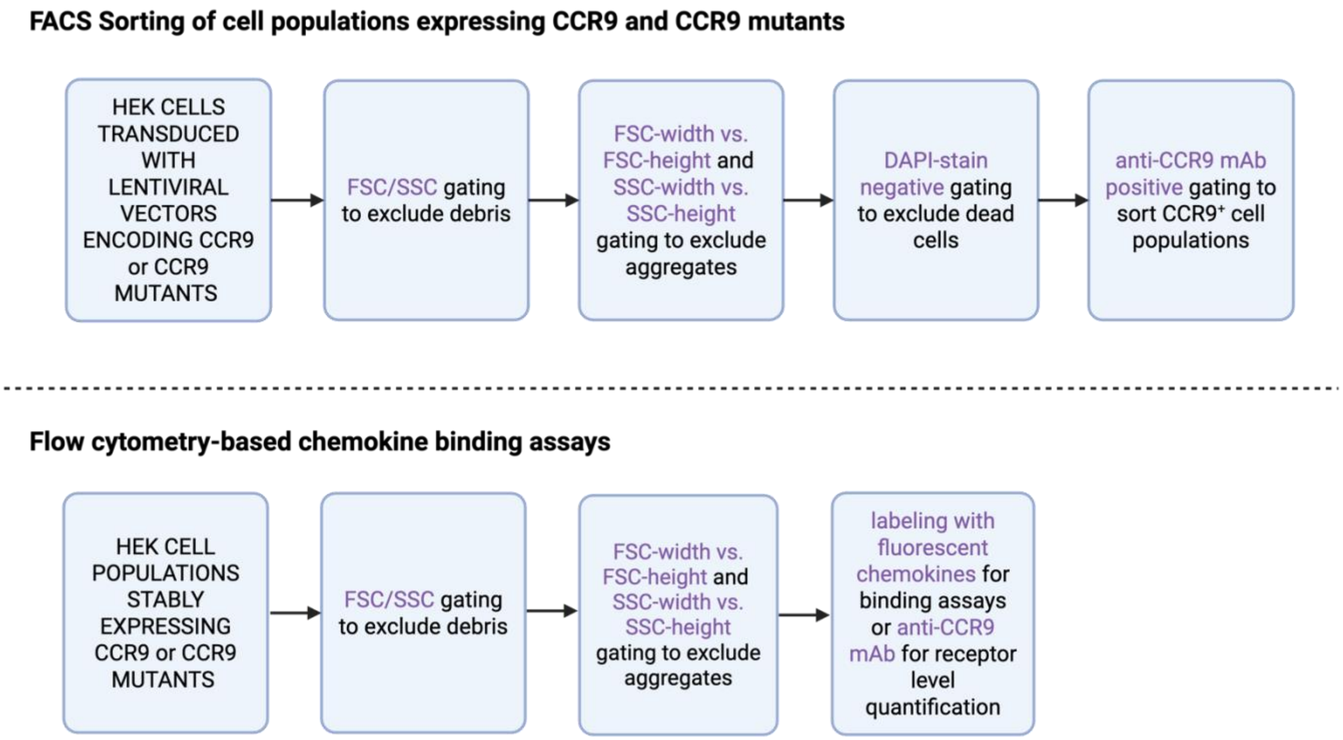

Supplementary Figure 32. Gating strategies used for FACS sorting and flow cytometry binding assays used in this study

Figure created in https://BioRender.com.

Supplementary Tables

Supplementary Table 1. Statistical analysis of the binding and functional assays on HEK293 cells expressing WT or mutant CCR9 cells and treated with CCL25.

Relates to **Figs. 2,3,4,5** and **Supplementary Figs. 9-12, 14,15.**

CCL25	300 nM CCL25 binding ratio to WT (eq. 1)			Ca^{2+} flux AUCRC ratio to WT		Arr3 recruitment AUCRC ratio to WT		
**Condition**	**mean±SEM (N)**	**p-value vs WT CCR9**	**p-value vs parental**	**mean±SEM (N)**	**p-value vs WT**	**mean±SEM (N)**	**p-value vs WT**	**Figs**
**WT**	1.00 ± 0.00 (5)	**n/a**	<0.0001	1.00 ± 0.00 (3)	**n/a**	1.00 ± 0.00 (3)	**n/a**	
**parental**	0.50 ± 0.05 (5)	<0.0001	**n/a**	**n/a**	**n/a**	**n/a**	**n/a**	
K40A	0.84 ± 0.08 (3)	0.545	0.001	0.10 ± 0.01 (3)	<0.0001	0.12 ± 0.02 (3)	<0.0001	2, 3, Suppl 9, Suppl 10
R44A	0.58 ± 0.07 (3)	0.000	0.545	0.14 ± 0.02 (3)	<0.0001	0.09 ± 0.02 (3)	<0.0001	
Y202A	0.81 ± 0.07 (2)	0.545	0.007	0.12 ± 0.02 (3)	<0.0001	0.19 ± 0.03 (3)	<0.0001	
K211A	1.08 ± 0.07 (3)	0.877	<0.0001	0.07 ± 0.01 (3)	<0.0001	0.08 ± 0.04 (3)	<0.0001	
S292A	0.66 ± 0.05 (2)	0.028	0.234	0.48 ± 0.10 (3)	0.002	0.87 ± 0.10 (3)	0.508	
D296A	0.84 ± 0.03 (3)	0.545	0.001	0.14 ± 0.04 (3)	<0.0001	0.45 ± 0.04 (3)	0.006	
S207A	1.59 ± 0.15 (3)	0.004	<0.0001	1.80 ± 0.06 (3)	0.018	1.65 ± 0.22 (3)	0.124	2, 4, Suppl 11, Suppl 12
T208A	0.95 ± 0.04 (3)	0.877	<0.0001	0.96 ± 0.10 (3)	0.794	0.06 ± 0.01 (3)	<0.0001	
Y126A	2.52 ± 0.43 (3)	<0.0001	<0.0001	2.05 ± 0.64 (3)	0.014	2.01 ± 0.26 (3)	0.021	2, 5, Suppl 14, Suppl 15
Q267A	2.06 ± 0.26 (2)	0.000	<0.0001	1.44 ± 0.38 (3)	0.325	0.29 ± 0.06 (3)	<0.0001	
N271A	1.06 ± 0.04 (2)	0.877	<0.0001	0.18 ± 0.01 (3)	<0.0001	2.41 ± 0.44 (3)	0.005	
F299A	2.23 ± 0.24 (2)	<0.0001	<0.0001	1.30 ± 0.29 (3)	0.467	1.52 ± 0.16 (3)	0.158	

Supplementary Table 2. Statistical analysis of the Ca^{2+} flux assay on MOLT-4 cells with WT CCL25 and the indicated CCL25 mutants.

Z denotes pyroglutamate (pGlu), incorporated during chemical synthesis. Concentration response curves were fitted with a four-parameter nonlinear regression using log-transformed concentrations; mutant models were compared one-by-one to the WT model using an F-test, followed by a Bonferroni correction for multiple testing [4]. The non-linear regression fit parameters obtained by merging two independent experiments are shown. Relates to **Fig. 6** and **Supplementary Fig. 20**.

CCL25 ala-scan	Ca^{2+} flux			
**Conditions**	**EC50 (nM)**	**Emax**	**Hill slope**	**p value vs WT CCL25**
**CCL25 WT**	26.64	0.63	1.09	**n/a**
CCL25-Z1A	23.59	0.64	1.20	ns
CCL25-G2A	13.04	0.68	1.27	0.017
CCL25-V3A	12.05	0.66	1.18	0.005
CCL25-F4A	22.59	0.63	1.19	ns
CCL25-E5A	16.65	0.62	1.35	ns
CCL25-D6A	46.47	0.62	1.06	<0.0015
CCL25-I28A	61.74	0.71	0.97	0.008
CCL25-Q29A	123.50	0.71	1.28	<0.0015
CCL25-E30A	30.97	0.79	0.81	0.062
CCL25-V31A	217.30	0.58	1.43	<0.0015
CCL25-S32A	464.70	0.49	1.56	<0.0015
CCL25-G33A	14.03	0.83	0.61	<0.0015
CCL25-S34A	80.75	0.73	1.15	0.002
CCL25-N36A	274.70	0.43	1.62	<0.0015
CCL25-L37A	132.10	0.66	1.10	<0.0015

Supplementary Table 3. Activity and sequences of the CCL25 N-terminally extended and natural-length analogs discovered by phage display.

Z denotes pyroglutamate (pGlu), incorporated during chemical synthesis. Agonist mode stimulation indexes (SIs) correspond to AUCRC of Ca^{2+} flux responses of MOLT-4 cells to CCL25 analogs across a range of concentrations. Antagonist mode SIs correspond to the AUCRC of Ca^{2+} flux responses of MOLT-4 cells to 100 nM CCL25 added 5 min after the addition of the CCR9 ligand in agonist mode. Refer to **Supplementary Fig. 21** legend for details.

				N-terminal sequence						
Compound	Agonist SI ± SEM	Antagonist SI ± SEM	№ independent experiments	0	1	2	3	4	5	6
CCL25	1	0	28	-	Z	G	V	F	E	D
Vercirnon	0.026 ± 0.003	0.508 ± 0.097	23							
CCL25-1P01	0.034 ± 0.02	1.165 ± 0.08	3	-	Z	G	A	L	R	Q
CCL25-1P02	0.24 ± 0.038	1.066 ± 0.107	3	-	Z	G	V	A	R	N
CCL25-1P03	0.095 ± 0.042	0.866 ± 0.178	3	-	Z	G	V	A	R	R
CCL25-1P04	0.053 ± 0.018	1.009 ± 0.121	3	-	Z	G	V	Q	R	I
CCL25-1P05	0.078 ± 0.005	1.011 ± 0.128	3	-	-	Z	L	G	V	Q
CCL25-1P06	1.221 ± 0.053	-0.304 ± 0.094	6	-	Y	Q	A	S	E	D
CCL25-1P07	0.166 ± 0.029	0.846 ± 0.163	3	-	Y	Q	S	R	E	D
CCL25-1P08	0.135 ± 0.015	0.877 ± 0.126	3	-	Y	S	Q	R	E	D
CCL25-1P09	0.113 ± 0.025	0.877 ± 0.12	3	Z	G	A	F	Q	P	D
CCL25-1P10	0.387 ± 0.025	0.628 ± 0.138	3	Z	G	G	F	K	Q	D
CCL25-1P11	0.191 ± 0.036	0.75 ± 0.114	3	Z	G	G	F	Q	W	D
CCL25-1P12	0.609 ± 0.017	0.308 ± 0.108	3	Z	G	F	L	T	A	D
CCL25-1P13	0.637 ± 0.07	0.226 ± 0.059	3	Z	G	G	L	Q	F	D
CCL25-1P14	0.392 ± 0.035	0.469 ± 0.097	3	Z	G	L	L	Q	Q	D
CCL25-1P15	0.381 ± 0.025	0.562 ± 0.139	3	Z	G	Q	L	Q	F	D
CCL25-1P16	0.295 ± 0.017	0.68 ± 0.152	3	K	D	L	Q	F	E	D
CCL25-1P17	0.471 ± 0.05	0.459 ± 0.094	3	L	D	A	Q	F	E	D
CCL25-1P18	0.656 ± 0.061	0.297 ± 0.126	3	T	D	I	Q	F	E	D
CCL25-1P19	0.55 ± 0.056	0.388 ± 0.075	3	V	D	G	Q	F	E	D
CCL25-1P20	0.652 ± 0.033	0.306 ± 0.087	3	V	E	L	Q	F	E	D
CCL25-1P21	0.334 ± 0.055	0.621 ± 0.04	3	E	F	L	R	F	E	D
CCL25-1P22	0.284 ± 0.018	0.726 ± 0.087	3	G	Q	L	K	F	E	D
CCL25-1P23	0.405 ± 0.01	0.722 ± 0.227	2	I	T	Q	R	F	E	D
CCL25-1P24	0.387 ± 0.009	0.674 ± 0.165	2	S	I	Q	R	F	E	D
CCL25-1P25	0.657 ± 0.068	0.423 ± 0.087	2	Z	G	I	Q	F	I	D
CCL25-1P26	0.542 ± 0.05	0.472 ± 0.033	2	Z	G	I	Q	W	I	D
CCL25-1P28	0.124 ± 0.014	0.968 ± 0.102	3	Z	G	I	W	Q	Y	D
CCL25-1P29	1.195 ± 0.06	-0.236 ± 0.11	2	Z	G	V	Q	Y	G	D
CCL25-1P30	0.459 ± 0.084	0.477 ± 0.031	3	Z	L	L	W	F	E	D
CCL25-1P31	0.051 ± 0.006	1.034 ± 0.094	3	Z	G	D	I	Q	P	D

CCL25-1P32	0.037 ± 0.009	0.925 ± 0.111	3	Z	G	D	Q	P	I	D
CCL25-1P33	0.076 ± 0.015	1.064 ± 0.135	3	-	R	G	R	Q	E	D
CCL25-1P34	0.082 ± 0.007	0.971 ± 0.07	3	-	R	R	A	E	E	D
CCL25-1P35	0.095 ± 0.017	0.996 ± 0.099	3	-	R	R	K	Q	E	D
CCL25-1P36	0.145 ± 0.024	1.057 ± 0.11	2	-	Z	G	K	S	Q	G
CCL25-1P37	0.063 ± 0.01	0.999 ± 0.121	3	-	Z	G	R	Q	A	Q
CCL25-1P38	0.074 ± 0.015	1.037 ± 0.144	3	-	Z	G	R	S	Q	Q
CCL25-1P39	0.141 ± 0.006	0.985 ± 0.018	2	-	Z	S	K	R	E	D
CCL25-1P40	0.377 ± 0.044	0.665 ± 0.041	2	-	Z	Y	K	Q	E	D
CCL25-1P41	0.074 ± 0.046	1.159 ± 0.139	2	-	Z	G	A	W	W	R
CCL25-1P42	0.064 ± 0.049	1.129 ± 0.147	2	-	Z	G	E	L	H	Q
CCL25-1P44	0.308 ± 0.005	0.904 ± 0.185	2	-	Z	G	Q	W	S	G
CCL25-1P45	0.129 ± 0.046	1.021 ± 0.171	2	Z	G	Q	Y	L	D	D
CCL25-1P46	0.532 ± 0.042	0.537 ± 0.137	2	Z	G	S	Q	L	Q	D
CCL25-1P47	0.191 ± 0.024	0.907 ± 0.06	2	G	R	D	Q	F	E	D
CCL25-1P48	0.13 ± 0.024	0.987 ± 0.169	2	G	R	E	Q	F	E	D
CCL25-1P49	0.755 ± 0.06	0.329 ± 0.208	2	-	V	F	Q	L	E	D
CCL25-1P50	0.204 ± 0.044	0.927 ± 0.145	2	-	V	Q	R	L	E	D

Supplementary Table 4. Statistical analysis of the binding and functional assays on HEK293 cells expressing WT or mutant CCR9 cells and treated with [1P6]CCL25.

Relates to **Fig. 8** and **Supplementary Figs. 23** to **29.**

[1P6] CCL25	300 nM [1P6] CCL25 binding ratio to WT (eq. 1)			Ca^{2+} flux AUCRC ratio to WT		Arr3 recruitment AUCRC ratio to WT	
**Condition**	**mean ± SEM (N)**	**p-value vs WT CCR9**	**p-value vs parental**	**mean ± SEM (N)**	**p-value vs WT**	**mean ± SEM (N)**	**p-value vs WT**
**WT**	1.00 ± 0.00 (5)	**n/a**	<0.0001	1.00 ± 0.00 (3)	**n/a**	1.00 ± 0.00 (3)	**n/a**
**parental**	0.24 ± 0.04 (5)	<0.0001	**n/a**	n/a ± n/a	**n/a**	n/a ± n/a	**n/a**
K40A	0.62 ± 0.04 (3)	0.006	<0.0001	0.26 ± 0.02 (3)	<0.0001	0.44 ± 0.01 (3)	<0.0001
R44A	0.59 ± 0.01 (3)	0.004	<0.0001	0.57 ± 0.03 (3)	<0.0001	0.59 ± 0.03 (3)	0.0004
Y202A	0.55 ± 0.03 (2)	0.005	0.0048	0.06 ± 0.00 (3)	<0.0001	0.53 ± 0.06 (3)	<0.0001
K211A	0.49 ± 0.08 (3)	0.000	0.0003	0.06 ± 0.00 (3)	<0.0001	0.15 ± 0.01 (3)	<0.0001
S292A	0.49 ± 0.10 (2)	0.001	0.002	0.59 ± 0.12 (3)	<0.0001	0.94 ± 0.01 (3)	0.727
D296A	0.47 ± 0.01 (3)	0.000	0.0003	0.26 ± 0.02 (3)	<0.0001	0.65 ± 0.06 (3)	0.003
S207A	1.49 ± 0.15 (3)	0.064	<0.0001	1.10 ± 0.03 (3)	0.597	1.12 ± 0.11 (3)	0.727
T208A	1.00 ± 0.04 (3)	0.795	<0.0001	0.99 ± 0.05 (3)	0.920	0.04 ± 0.01 (3)	<0.0001
Y126A	2.15 ± 0.44 (3)	0.000	<0.0001	1.19 ± 0.04 (3)	0.383	1.11 ± 0.06 (3)	0.727
Q267A	1.08 ± 0.07 (2)	0.767	<0.0001	0.76 ± 0.05 (3)	0.065	0.54 ± 0.01 (3)	<0.0001
N271A	1.09 ± 0.08 (2)	0.767	<0.0001	0.51 ± 0.06 (3)	<0.0001	1.45 ± 0.05 (3)	0.013
F299A	1.58 ± 0.04 (2)	0.019	<0.0001	0.89 ± 0.06 (3)	0.571	1.36 ± 0.06 (3)	0.038

Supplementary Table 5. Key resources and reagents.

Reagent name	Manufacturer	Catalog number
Recombinant CCL25(1-127)	R&D Systems	9046-TK
TAMRA-PEG4-DBCO (Dibenzocyclooctyne-PEG4-Fluor 545)	Sigma-Aldrich	760773
Amicon Ultra-0.5 Centrifugal Filter 10 kDa MWCO	Millipore	UFC5010
Alexa Fluor 647-conjugated Mouse Anti-Human CD199 (CCR9) mAb	BD Biosciences	557975; RRID: AB_2073270
HEK293T cells	ATCC	CRL-3216
MOLT-4 cells	ATCC	CRL-1582
DMEM, high glucose, GlutaMAX™ Supplement, pyruvate	Thermo Fisher Scientific	31966021
RPMI 1640 Medium, GlutaMAX™ Supplement	Thermo Fisher Scientific	61870036
Fetal bovine serum (FBS)	Thermo Fisher Scientific	A5256701
FluoroBrite™ DMEM	Thermo Fisher Scientific	A1896701
Penicillin-Streptomycin (10,000 U/mL)	Thermo Fisher Scientific	15140122
JetPRIME® transfection reagent	Polyplus	101000027
Coelenterazine-h (CTZ-h)	Carbosynth	FC36332
Screen Quest™ Fluo-8 No Wash Ca^{2+} Assay Kit	AAT BioQuest Lubio Science	36316

Supplementary Table 6. Constructs and cloning

Construct	Plasmid backbone	Source lab	References
Gαi1(91)-RLuc2	pcDNA3.1(+)	Michel Bouvier, Univ. of Montreal	Quoyer et al. 2013
mVenus-Gβ1	pcDNA3.1	Nevin A. Lambert, Univ. of Augusta	Brown et al. 2016; Hollins et al. 2009
Gγ2	pcDNA3.1	Aska Inoue, Tohoku Univ.	Inoue et al. 2019
FUGW-CCR9A WT and mutants	FUGW	This work	
FUGW-CCR9A-RLuc8 WT and mutants	FUGW	This work	
FUGW-YFP-arrestin 3	FUGW	Oliver Hartley, Univ. of Geneva	Martins et al. 2020
psPAX2	psPAX2	Patrick Salmon, Univ. of Geneva	Giry-Laterrière et al. 2011
pMD2G	pMD2G	Patrick Salmon, Univ. of Geneva	Giry-Laterrière et al. 2011

Supplementary References

1. I Kufareva, M Rueda, V Katritch, RC Stevens and R Abagyan. Status of GPCR modeling and docking as reflected by community-wide GPCR Dock 2010 assessment. *Structure* **19**, 1108-26 (2011)
2. I Kufareva, V Katritch, RC Stevens and R Abagyan. Advances in GPCR modeling evaluated by the GPCR Dock 2013 assessment: meeting new challenges. *Structure* **22**, 1120-1139 (2014)
3. R Chitsazi, Y Wu, GD participants, RC Stevens, S Zhao and I Kufareva. The 4th GPCR Dock: assessment of blind predictions for GPCR-ligand complexes in the era of AlphaFold. *bioRxiv*, 2025.04.18.647407 (2025)
4. H Abdi. Bonferroni and Šidák corrections for multiple comparisons. *Encyclopedia of measurement and statistics* **3**, 2007 (2007)
