# Supplementary material for: Noncanonical roles of chemokine regions in CCR9 activation revealed by structural modeling and mutational mapping
Source: Nat Commun. 2025 Aug 18;16:7695. doi: 10.1038/s41467-025-62321-9 (PMC12361432; doi:10.1038/s41467-025-62321-9)
Supplement: Supplementary file 2 — Description of Additional Supplementary Files [file 41467_2025_62321_MOESM2_ESM.pdf]

Description of Additional Supplementary Files

File Name: Supplementary Data 1
Description: **3D coordinates of CCR9 chemokine models shown in the manuscript figures, in the PDB format.**
For the 'Fig4HIJ' model ensemble, the B-factor field contains the 'degree of activation' measured as the distance between CCR9 Y126/oh and N271/nd2. For 'Fig7' models, the B-factor field contains RTCNN scores projected onto chemokine residue backbone and side chains. The models belong to the initial 5x5 ensembles constructed with AF2.

File Name: Supplementary Data 2
Description: **500x additional AF2 models of the CCR9-CCL25 complex.**
Models were constructed with the same means as the original 5x5 AF2 model ensemble but with a larger number of random seeds.

File Name: Supplementary Data 3
Description: **100x AF3 models of the CCR9-CCL25 complex, and Terms of Use.** Models were constructed using AlphaFold server (https://alphafoldserver.com/) and 20 random seeds.

File Name: Supplementary Data 4
Description: **Interactive ICM Browser sessions for the molecular figures in the manuscript.**
Files can be opened and manipulated in the free ICM Browser, downloadable from https://www.molsoft.com/icm_browser.html.

File Name: Supplementary Data 5
Description: **100x AF3 models of the CCR9-[1P6]CCL25 complex, and Terms of Use.** Models were constructed using AlphaFold server (https://alphafoldserver.com/) and 20 random seeds.
