# Supplementary material for: Noncanonical roles of chemokine regions in CCR9 activation revealed by structural modeling and mutational mapping
Source: Nat Commun. 2025 Aug 18;16:7695. doi: 10.1038/s41467-025-62321-9 (PMC12361432; doi:10.1038/s41467-025-62321-9)
Supplement: Supplementary file 8 — Reporting Summary [file 41467_2025_62321_MOESM8_ESM.pdf]

nature portfolio

Corresponding author(s): Oliver Hartley, Irina Kufareva

Last updated by author(s): Mar 23, 2025

Reporting Summary

Nature Portfolio wishes to improve the reproducibility of the work that we publish. This form provides structure for consistency and transparency in reporting. For further information on Nature Portfolio policies, see our Editorial Policies and the Editorial Policy Checklist.

Statistics

For all statistical analyses, confirm that the following items are present in the figure legend, table legend, main text, or Methods section.

n/a	Confirmed	
☐	☒	The exact sample size (*n*) for each experimental group/condition, given as a discrete number and unit of measurement
☐	☒	A statement on whether measurements were taken from distinct samples or whether the same sample was measured repeatedly
☐	☒	The statistical test(s) used AND whether they are one- or two-sided<br>*Only common tests should be described solely by name; describe more complex techniques in the Methods section.*
☐	☒	A description of all covariates tested
☐	☒	A description of any assumptions or corrections, such as tests of normality and adjustment for multiple comparisons
☐	☒	A full description of the statistical parameters including central tendency (e.g. means) or other basic estimates (e.g. regression coefficient) AND variation (e.g. standard deviation) or associated estimates of uncertainty (e.g. confidence intervals)
☐	☒	For null hypothesis testing, the test statistic (e.g. *F*, *t*, *r*) with confidence intervals, effect sizes, degrees of freedom and *P* value noted<br>*Give P values as exact values whenever suitable.*
☒	☐	For Bayesian analysis, information on the choice of priors and Markov chain Monte Carlo settings
☒	☐	For hierarchical and complex designs, identification of the appropriate level for tests and full reporting of outcomes
☒	☐	Estimates of effect sizes (e.g. Cohen's *d*, Pearson's *r*), indicating how they were calculated

Our web collection on statistics for biologists contains articles on many of the points above.

Software and code

Policy information about availability of computer code

Data collection	AlphaFold2 Multimer v2.3.2 (Google DeepMind) - used for modeling CCR9 complexes with chemokines<br>AlphaFold3 server (Google DeepMind / isomorphic Labs) - used for generating of additional CCR9 complex models with chemokines<br>ICM Pro 3.9-3b (Molsoft LLC) - used for refinement of AF2-generated CCR9 complex models with chemokines
Data analysis	Prism 10.0 (GraphPad) - used for normalization, collation, statistical analyses, and visualization of experimental data<br>ICM Pro 3.9-3b (Molsoft LLC) - used for the analysis and visualization of CCR9 complex models with chemokines<br>CytExpert (Beckman Coulter) - used for the analysis of flow cytometry data<br>Python 3.12.2 and matplotlib 3.8.3 - used for the generation of radar plots

For manuscripts utilizing custom algorithms or software that are central to the research but not yet described in published literature, software must be made available to editors and reviewers. We strongly encourage code deposition in a community repository (e.g. GitHub). See the Nature Portfolio guidelines for submitting code & software for further information.

Data

Policy information about availability of data

All manuscripts must include a data availability statement. This statement should provide the following information, where applicable:

- Accession codes, unique identifiers, or web links for publicly available datasets
- A description of any restrictions on data availability
- For clinical datasets or third party data, please ensure that the statement adheres to our policy

All data needed to evaluate the conclusions in the study are provided in the main manuscript, Supplementary Information, Supplementary Data, and Source Data files.

Research involving human participants, their data, or biological material

Policy information about studies with human participants or human data. See also policy information about sex, gender (identity/presentation), and sexual orientation and race, ethnicity and racism.

Reporting on sex and gender	*Use the terms sex (biological attribute) and gender (shaped by social and cultural circumstances) carefully in order to avoid confusing both terms. Indicate if findings apply to only one sex or gender; describe whether sex and gender were considered in study design; whether sex and/or gender was determined based on self-reporting or assigned and methods used. Provide in the source data disaggregated sex and gender data, where this information has been collected, and if consent has been obtained for sharing of individual-level data; provide overall numbers in this Reporting Summary. Please state if this information has not been collected. Report sex- and gender-based analyses where performed, justify reasons for lack of sex- and gender-based analysis.*
Reporting on race, ethnicity, or other socially relevant groupings	*Please specify the socially constructed or socially relevant categorization variable(s) used in your manuscript and explain why they were used. Please note that such variables should not be used as proxies for other socially constructed/relevant variables (for example, race or ethnicity should not be used as a proxy for socioeconomic status). Provide clear definitions of the relevant terms used, how they were provided (by the participants/respondents, the researchers, or third parties), and the method(s) used to classify people into the different categories (e.g. self-report, census or administrative data, social media data, etc.) Please provide details about how you controlled for confounding variables in your analyses.*
Population characteristics	*Describe the covariate-relevant population characteristics of the human research participants (e.g. age, genotypic information, past and current diagnosis and treatment categories). If you filled out the behavioural & social sciences study design questions and have nothing to add here, write "See above."*
Recruitment	*Describe how participants were recruited. Outline any potential self-selection bias or other biases that may be present and how these are likely to impact results.*
Ethics oversight	*Identify the organization(s) that approved the study protocol.*

Note that full information on the approval of the study protocol must also be provided in the manuscript.

Field-specific reporting

Please select the one below that is the best fit for your research. If you are not sure, read the appropriate sections before making your selection.

☒ Life sciences ☐ Behavioural & social sciences ☐ Ecological, evolutionary & environmental sciences

For a reference copy of the document with all sections, see nature.com/documents/nr-reporting-summary-flat.pdf

Life sciences study design

All studies must disclose on these points even when the disclosure is negative.

Sample size	Technical replicates (n=3) were performed at each tested ligand concentration for the binding and signaling assays described in this study.
Data exclusions	No data was excluded from the analysis.
Replication	As noted in the manuscript, results of three independent experiments are presented for all of the signaling assays. For certain binding assays only two independent experimental replicates were performed owing to limited available quantities of fluorescently labeled chemokines. Data from all experimental replicates are provided in the Supplementary Materials.
Randomization	Randomization was not applicable, as there were no treatment groups or animal/human subjects.
Blinding	Blinding was not relevant to this study as no group allocation was involved; all experiments were conducted using defined molecular constructs and in vitro systems.

Reporting for specific materials, systems and methods

We require information from authors about some types of materials, experimental systems and methods used in many studies. Here, indicate whether each material, system or method listed is relevant to your study. If you are not sure if a list item applies to your research, read the appropriate section before selecting a response.

Materials & experimental systems

n/a	Involved in the study
☐	☒ Antibodies
☐	☒ Eukaryotic cell lines
☒	☐ Palaeontology and archaeology
☒	☐ Animals and other organisms
☒	☐ Clinical data
☒	☐ Dual use research of concern
☒	☐ Plants

Methods

n/a	Involved in the study
☒	☐ ChIP-seq
☐	☒ Flow cytometry
☒	☐ MRI-based neuroimaging

Antibodies

Antibodies used	Alexa Fluor 647-conjugated Mouse Anti-Human CD199 (CCR9) mAb; BD Biosciences 557975; RRID: AB_2073270 dilution 1:100
Validation	This antibody has been validated by BD biosciences for use in flow cytometry.

Eukaryotic cell lines

Policy information about cell lines and Sex and Gender in Research

Cell line source(s)	MOLT-4: ATCC, CRL-1582<br>CHO-K1: ATCC, CCL-61<br>HEK293T: ATCC, CRL-3216
Authentication	Since the lines were obtained directly form ATCC they were not further authenticated
Mycoplasma contamination	All cell lines used tested negative for mycoplasma before banking.
Commonly misidentified lines (See ICLAC register)	All cell lines used (HEK and MOLT-4) were sourced from ATCC and are not on the ICLAC list of misidentified lines.

Plants

Seed stocks	NA
Novel plant genotypes	NA
Authentication	NA

Flow Cytometry

Plots

Confirm that:

☐ The axis labels state the marker and fluorochrome used (e.g. CD4-FITC).

☐ The axis scales are clearly visible. Include numbers along axes only for bottom left plot of group (a 'group' is an analysis of identical markers).

☐ All plots are contour plots with outliers or pseudocolor plots.

☐ A numerical value for number of cells or percentage (with statistics) is provided.

Methodology

Sample preparation	In all cases cells were detached with 0.5 mM EDTA; 200,000 cells per sample were incubated with fluorescently labeled chemokines or antibody diluted in FACS buffer (1x PBS, 1 mM EDTA, 1% BSA).
Instrument	BD FACS Aria® Fusion<br>CytoFLEX (Beckman Coulter)
Software	CytExpert (Beckman Coulter)
Cell population abundance	Stability of receptor expression on cell lines expressing CCR9 and CCR9 mutants wasroutinely controlled using the Alexa Fluor 647-conjugated Mouse Anti-Human CD199 (CCR9) mAb; BD Biosciences 557975; RRID: AB_2073270.
Gating strategy	For cell sorting, mononuclear cells were first gated based on forward and side scatter (FSC/SSC) parameters to exclude debris. Single cells were identified using FSC-width vs. FSC-height and SSC-width vs. SSC-height to eliminate aggregates. Live single cells were subsequently gated as the DAPI-negative population, from which CCR9^{+} cells were selected.<br>For flow cytometry binding assays, mononuclear cells were gated by FSC/SSC parameters without DAPI staining, and binding was assessed directly.

☐ Tick this box to confirm that a figure exemplifying the gating strategy is provided in the Supplementary Information.

nature portfolio | reporting summary
