# Supplementary material for: Noncanonical roles of chemokine regions in CCR9 activation revealed by structural modeling and mutational mapping
Source: Nat Commun. 2025 Aug 18;16:7695. doi: 10.1038/s41467-025-62321-9 (PMC12361432; doi:10.1038/s41467-025-62321-9)
Supplement: Supplementary file 10 — Source Data [file 41467_2025_62321_MOESM10_ESM.zip › New folder/Hartley source data list.docx]

**Source Data 1:** Source data for Ca^2+^ flux data presented in this study.

**Source Data 2:** Source data for BRET-based arrestin recruitment data presented in this study.

**Source Data 3:** Source data for flow cytometry-based chemokine and antibody binding data presented in this study.
